# Local Oestrogen for Pelvic Floor Disorders: A Systematic Review

**DOI:** 10.1371/journal.pone.0136265

**Published:** 2015-09-18

**Authors:** M. A. Weber, M. H. Kleijn, M. Langendam, J. Limpens, M. J. Heineman, J. P. Roovers

**Affiliations:** 1 Department of Obstetrics and Gynaecology, Academic Medical Center, Amsterdam, the Netherlands; 2 Department of Clinical Epidemiology, Biostatistics and Bioinformatics, Academic Medical Center, Amsterdam, the Netherlands; 3 Medical Library, Academic Medical Center, Amsterdam, the Netherlands; Cedars-Sinai Medical Center, UNITED STATES

## Abstract

**Objective:**

The decline in available oestrogen after menopause is a possible etiological factor in pelvic floor disorders like vaginal atrophy (VA), urinary incontinence (UI), overactive bladder (OAB) and pelvic organ prolapse (POP). This systematic review will examine the evidence for local oestrogen therapy in the treatment of these pelvic floor disorders.

**Evidence Acquisition:**

We performed a systematic search in MEDLINE, EMBASE, the Cochrane Central Register of Controlled Trials and the non-MEDLINE subset of PubMed from inception to May 2014. We searched for local oestrogens and VA (I), UI/OAB (II) and POP (III). Part I was combined with broad methodological filters for randomized controlled trials (RCTs) and secondary evidence. For part I and II two reviewers independently selected RCTs evaluating the effect of topical oestrogens on symptoms and signs of VA and UI/OAB. In part III all studies of topical oestrogen therapy in the treatment of POP were selected. Data extraction and the assessment of risk of bias using the Cochrane Risk of Bias Tool was undertaken independently by two reviewers.

**Evidence Synthesis:**

The included studies varied in ways of topical application, types of oestrogen, dosage and treatment durations. Objective and subjective outcomes were assessed by a variety of measures. Overall, subjective and urodynamic outcomes, vaginal maturation and vaginal pH changed in favor of vaginal oestrogens compared to placebo. No obvious differences between different application methods were revealed. Low doses already seemed to have a beneficial effect. Studies evaluating the effect of topical oestrogen in women with POP are scarce and mainly assessed symptoms and signs associated with VA instead of POP symptoms.

**Conclusion:**

Topical oestrogen administration is effective for the treatment of VA and seems to decrease complaints of OAB and UI. The potential for local oestrogens in the prevention as well as treatment of POP needs further research.

## Introduction

Oestrogen is of great importance in the function of the genital and lower urinary tract with oestrogen receptors being present in the bladder, urethra, vagina, and pelvic floor musculature [1].

Oestrogen receptors play a role in the supportive mechanism of the pelvis by controlling the synthesis and breakdown of collagen [2]. Also, the tissues of the female urinary continence mechanism are sensitive to oestrogen. Oestrogens may affect continence by enhancing urethral resistance by increasing the number of periurethral vessels that account for one-third of urethral pressure [3]. Moreover, oestrogens can reduce the frequency and amplitude of detrusor contractions and so raise the sensory threshold of the bladder and promote relaxation of the detrusor muscle [4,5].

For these reasons the decline in available oestrogen after menopause is a possible etiological factor for pelvic floor disorders. Pelvic floor disorders include stress urinary incontinence (SUI), urge urinary incontinence (UUI) and pelvic organ prolapse (POP) and together they are estimated to occur in up to 40% of postmenopausal women [6,7]. Half of all postmenopausal women are thought to suffer from vaginal atrophy (VA), which commonly causes symptoms such as vaginal dryness, irritation or itching, dyspareunia and thin and frail epithelia [8].

In the past, studies have focused on systemic hormone replacement therapies, more recently topical oestrogens have become the focus of interest in the treatment of pelvic floor disorders as this reduces adverse effects. Treatment with topical oestrogens (in the form of tablets, pessaries, creams, and the oestradiol-releasing vaginal ring) have proven to be effective for the symptoms associated with vaginal atrophy. A review of 19 randomized controlled trials (RCTs) including 4162 women concluded that the available topical oestrogens are equally effective in relation to each other for treating VA and associated symptoms [9]. However, additional trials providing long-term data were advised. Moreover, a large trial variation existed, with small sample sizes and diversity in the outcomes measuring efficacy, safety and tolerance of the use of local oestrogen in vaginal atrophy [9].

In the treatment of urinary incontinence different options are available, including pelvic floor muscle training, antimuscarinic medications (for urge urinary incontinence) and surgery (for stress urinary incontinence). Oestrogen has been used to treat incontinence over a number of years, either alone or in combination with some of these other options, and there is evidence that urinary incontinence may improve with local oestrogen treatment [10]. In contrast, systemic hormone replacement therapy seems to worsen urinary incontinence [11]. The possible worsening of urinary incontinence with systemic oestrogen therapy as well as the concerns about adverse effects of systemic treatment (for example regarding breast cancer, effects on endometrium or thromboembolic diseases), makes further evaluation of local oestrogen therapy in the treatment of urinary incontinence of great value. The currently available evidence has to be interpreted with caution because the treatment effects are based on a relatively low number of patients and a wide range of types, dosages and duration of oestrogen treatment [10]. Moreover, also in the studies regarding local oestrogen treatment for urinary tract symptoms, there is a diversity in the outcomes measured (urodynamic or clinical) and populations studied [12].

The available evidence regarding vaginal oestrogen therapy in postmenopausal women with overactive bladder (OAB) symptoms (urinary urgency, frequency, nocturia, with or without urge urinary incontinence) is encouraging [13,14]. However, it is not clear if subjective improvement in OAB symptoms reflects a direct effect on lower urinary tract function or a indirect effect via reversing VA [15].

How oestrogen changes collagen metabolism related to POP is still unclear [16]. One hypothesis is that oestrogen brings the collagen metabolism back to a premenopausal state [17]. Consequently, oestrogen deficiency could weaken the supporting ligaments of the pelvic organs, as well as causing thinning of the vaginal epithelium [18]. These factors could contribute to POP. Oestrogens alone or together with other forms of treatment (i.e. vaginal pessaries, pelvic floor muscle training or surgery), may help in the treatment of POP by increasing synthesis of collagen and improving the strength of the vaginal epithelium. Evidence regarding the effectiveness of topical oestrogens in the treatment of POP is, however, lacking [19].

With this systematic review we provide a complete overview of the current evidence regarding topical oestrogen therapy in the treatment of pelvic floor disorders.

## Methods

### Eligibility criteria

#### Studies regarding vaginal atrophy

All randomised controlled trials of vaginally administered oestrogen for the treatment of symptoms of vaginal atrophy (as defined by the trialists) in postmenopausal women with a duration of treatment of at least 12 weeks.

#### Outcomes regarding vaginal atrophy

Subjective efficacy:

- Presence of vaginal dryness, vaginal itching/irritation and dyspareunia but also composite scores like:
  - Most bothersome symptom (MBS) approach: The MBS consists of a list of symptoms (most commonly the four symptoms of vaginal dryness, vaginal itching/irritation, dyspareunia and vaginal soreness). At baseline, participants address each of these symptoms as not present, mild, moderate, or severe and then select one symptom previously reported as moderate or severe as the MBS. The MBS is then re-evaluated at the end of treatment, and the change in severity is used to assess subjective improvement [20,21].
  - Urogenital score: includes the subjective assessment of symptoms like urinary frequency and urgency, dyspareunia and vaginal dryness[22].
  - Total score index of vaginal atrophy: reported from 0 to 3 according to the assessment of symptoms by the participant, in combination with the assessment of a physician according to gynaecologic examination (no symptoms: 0–0.50, minor symptoms: 0.51–1.00, moderate symptoms: 1.01–2.00, severe symptoms: 2.01–3.00) [23].
- Clinician assessment of the vaginal wall appearance including assessment of the presence or absence of petechiae on the vaginal wall, vaginal wall pallor, friability of the vaginal wall (defined as any bleeding occurring during examination), vaginal dryness but also composite scores like:
  - Vaginal physical examination scale: includes assessment of vaginal wall petechiae, vaginal wall friability, rugae and decreased vaginal wall elasticity (conization) [24].
  - Vaginal Health Index (VHI) / Vaginal Dryness Index: includes assessment of vaginal moisture, fluid volume, elasticity, epithelial integrity on a scale of 1 (poorest) to 5 (best), and vaginal pH [22,25–27].
  - Genital Health Clinical Evaluation (GHCE): the GHCE is a tool used to assess six parameters (vaginal fluid secretion, epithelium, colour, moisture, rugosity and pH) scored on a scale 1 to 4. A higher score indicates less atrophy [28].

Objective efficacy: vaginal pH measurement and cytological assessment including vaginal maturation index (VMI), vaginal maturation value (VMV) and karyopyknotic index (KPI or KI). VMI represents the percentage of parabasal, intermediate and superficial squamous cells appearing on a vaginal smear [29]. Cytomorphologically, VA can be defined as a condition with a very low percentage of superficial cells and a high percentage of (para)basal and intermediate cells [30]. In a formula the different cell types can be multiplied by certain factors to obtain the VMV. There is a variety in formulas to calculate the VMV [8]. KPI or KI is described as measuring the relationship of superficial cells to intermediate cells [31] but also as the percentage of superficial cells of the total amount of the squamous cells examined [32,33].

Safety: Adverse events related to treatment

#### Studies regarding urinary incontinence and overactive bladder symptoms

All randomised controlled trials of local oestrogen therapy for the treatment of overactive bladder symptoms, urinary stress, urge or mixed incontinence in postmenopausal women diagnosed by symptom classification or by urodynamic diagnosis, as defined by the trialists.

#### Outcomes regarding urinary incontinence and overactive bladder

Subjective efficacy: Patient reported symptoms of urinary incontinence, urgency and/or overactive bladder, improvement or cure of symptoms of urinary incontinence, urgency and/or overactive bladder, and disease specific quality of life questionnaire (Incontinence Impact Questionnaire (IIQ)).

Semi-objective efficacy: Frequency of micturitions and number of incontinent episodes (as indicated from bladder diary), pad tests (weight of urine loss) and pad changes.

Objective efficacy: Urodynamic measures including maximum bladder capacity (ml), maximum urethral closure pressure (MUCP) (cmH20) and volume at first urge to void (ml).

Safety: Adverse events related to treatment.

#### Studies regarding pelvic organ prolapse

All studies of local oestrogen therapy for the treatment of pelvic organ prolapse in postmenopausal women with any degree of pelvic organ prolapse.

#### Outcomes regarding pelvic organ prolapse

Subjective efficacy: patient reported symptoms of POP (sense of pressure or bulge vaginally, abdominal or back pain, urinary or bowel symptoms), improvement or cure of symptoms associated with POP, satisfaction with treatment outcome, postponement or no need for other treatments like pelvic floor muscle training, mechanical devices or surgery, disease-specific quality of life questionnaire (Urogenital Distress Inventory (UDI)) and clinicians observed improvement or cure of POP using the POP-Q system [34].

Safety: Adverse events related to treatment.

### Interventions

Oestrogenic preparations administered intra-vaginally, including creams, tablets, ovules/pessaries and rings.

### Search strategy

Our review followed the PRISMA (Preferred Reporting Items for Systematic Reviews and Meta-Analysis) guidelines for reporting. A medical information specialist (JL) performed a comprehensive search of MEDLINE and EMBASE (via the OVID-interface), CENTRAL, the non-MEDLINE subset of PubMed and prospective trial registers from inception to May the 25^th^ 2014. Both indexterms and text words were used, with no language or other restrictions.

The search consisted of three parts. The basis was an extensive search for local or vaginally applied oestrogens. This search was successively combined with a broad search for 1] vaginal atrophy (VA), 2] urinary incontinence/overactive bladder (UI/OAB) and 3] pelvic organ prolapse (POP). The VA–search, retrieving many randomized controlled trials (RCTs), was combined with a sensitive filter for RCTs (adapted from the Cochrane Collaboration). In addition we checked the availability of systematic reviews (SRs) by applying a methodological filter for secondary studies. No methodological filters were needed for part 2 and 3 (for the OVID MEDLINE search strategy see S1 Appendix).

We cross-checked the reference lists and the citing articles of the identified relevant papers and adapted the search in case of additional relevant studies. The results were entered and de-duplicated in Reference Manager (version 12.0).

### Data collection and analysis

Eligible studies were selected from the identified references by applying the inclusion criteria, first on title and abstract, and in a second step on full text.

If studies were sufficiently similar with regard to clinical aspects and study design, a pooled effect (fixed effect model) was calculated using Review Manager (version 5.2) software. In case of substantial heterogeneity a random effects model was used.

Studies that did not report their results in enough detail to allow data extraction (e.g. missing standard deviations, or presentation of the results in graphs) were not included in the appendices with the analyses. The results of these studies are described narratively in the Results section.

The effect measures were risk ratio (RR) for dichotomous data and mean difference (MD) for continuous data, with 95% confidence intervals (CI). Data analysis was performed using Review Manager software following the guidance the Cochrane Reviewers’ Handbook [35]

### Assessment of risk of bias

Risk of bias was assessed using the Cochrane Risk of Bias Tool [35]. We assessed the risk of bias of the studies that were published after the date of the search in the Cochrane reviews of Suckling [9], Cody [10] or Ismail [19] or studies that were not included in the Cochrane reviews but were considered relevant for our review. For the studies included in the Cochrane reviews we used the Cochrane author’s assessment of risk of bias. Selection, data extraction and risk of bias assessment were performed by two reviewers independently. Disagreements were resolved by discussion, or if necessary, by a third reviewer.

## Results

### Topical oestrogens to treat vaginal atrophy

#### Studies

**Fig 1:**
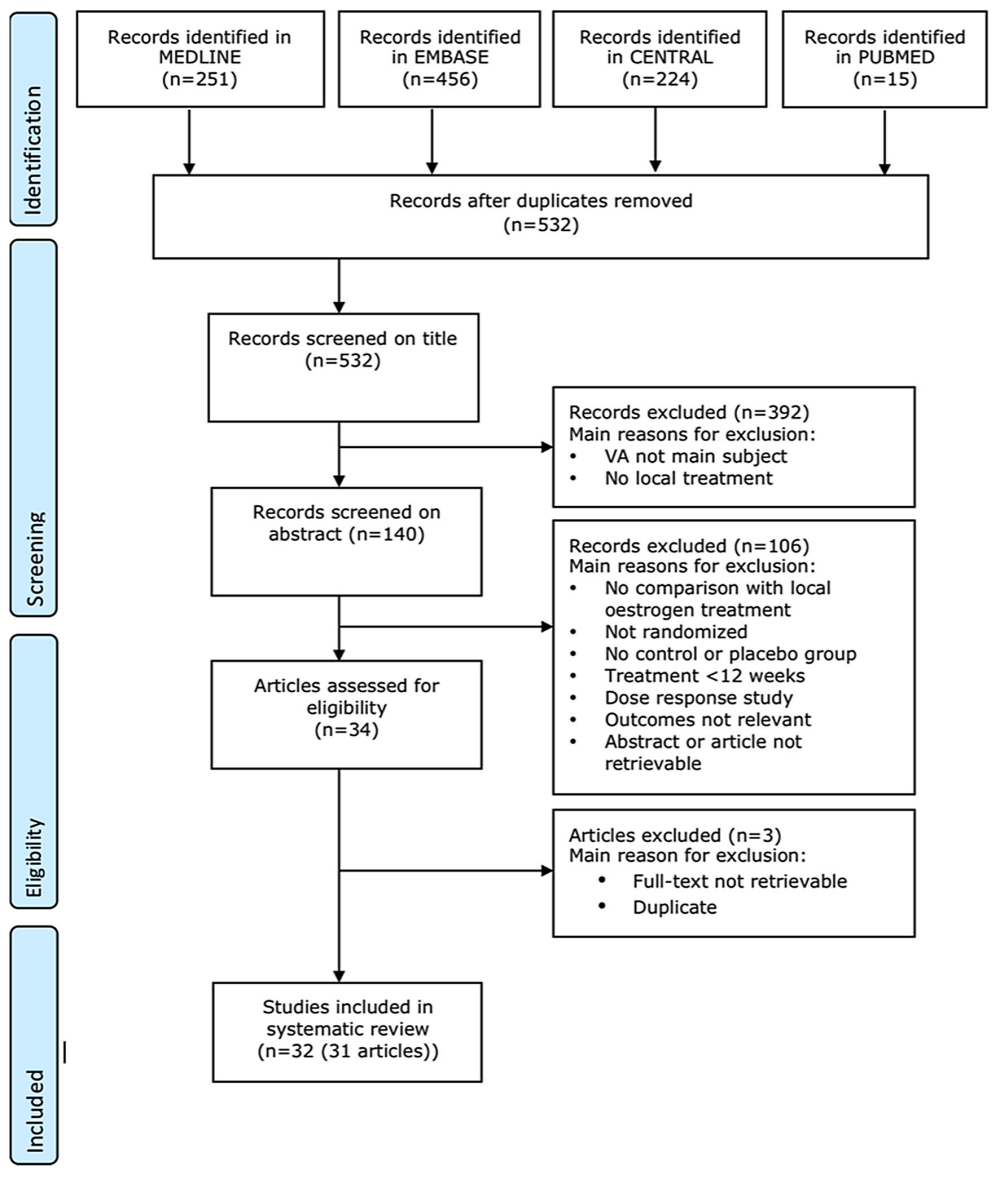
Flow chart local oestrogens for vaginal atrophy.

Our literature search considering topical oestrogen treatment for vaginal atrophy retrieved 532 studies. A total of 32 randomized controlled trials (31 articles) were selected for this review, of which 18 were also included in the Cochrane review of Suckling and co-workers [9]. Main reasons for exclusion and the flow of records through the selection process are provided in the flowchart (Fig 1).

As can be seen in Table 1, there was a wide variety of topical oestrogen administration forms. Local administration of oestrogen was compared to placebo or no treatment or non-hormonal treatment. Other studies compared different application forms, doses or combination treatment. The 32 trials included over 6500 participants. Treatment ranged from 12 weeks up to 12 months, 24 out of the 32 studies were included in the quantitative analysis. Eight studies did not report their data in sufficient detail to allow data extraction [36–43].

**Table 1:** Comparisons in studies regarding local oestrogen treatment for vaginal atrophy.

Intervention	Control	Studies
***Vaginal oestrogen versus placebo***		
Tablets/pessary 0.01–0.03 mg	Placebo	Bachmann et al. 2008; Eriksen et al. 1992;Jaisamrarn et al. 2013; Griesser et al. 2012; Simon et al. 2008; Simunic et al. 2003
Ovule 1mg	Placebo	Dessole et al. 2004
Cream 0,3–0.625mg	Placebo	Freedman et al. 2009; Raghunandan et al. 2010; Bachmann et al. 2009; Lima et al. 2013
Gel 0.05 mg	Placebo	Cano et al. 2012
Ring 0.0075 mg/24hr	Placebo	Casper et al. 1999 study 2; Speroff et al. 2003
Pessary 0,2 mg	Placebo	Griesser et al. 2012
Depot 3.5mg	Placebo	Foidart et al.1991
***Different application methods of vaginal oestrogen***		
Ring 0.0065–0.0095 mg/24h	Patch 0.014 mg	Gupta et al. 2008
	Tablet 0.025 mg	Weisberg et al. 2005
	Cream 0.5 mg	Barentsen et al. 1997; Ayton et al. 1996; Nachtigall et al. 1995
	Pessary 0.5 mg	Henriksson et al. 1994; Casper et al. 1999 study 1
Tablet 25 microgram	Cream 1gram / 1,25mg	Manonai et al. 2001; Rioux et al. 2000
***Vaginal oestrogen versus non-hormonal treatment***		
Cream 0.5–0.625 mg	Replens	Bygdeman et al. 1996; Nachtigall et al. 1994
***Different doses of vaginal oestrogen***		
Tablet 0.01 mg	Tablet 0.025 mg	Bachmann et al. 2008
Ring 0.10–0.14 mg	Ring 0.05–0.06 mg	Nash et al. 1999; Speroff et al. 2003
Promestriene 1% cream	Estriol 0.1% cream	Bruno et al. 2012
Tablet 25 microgram	Vagitory 0,5mg	Dugal et al. 2000
***Vaginal oestrogen versus combination therapy***		
Oestrogen cream 0.625 mg and 0.5 mg of 2% testosterone cream	Oestrogen cream 0.625 mg	Raghunandan et al. 2010
Ovule 1 mg and pelvic floor rehabilitation	Ovule 1 mg	Capobianco et al. 2012
Triple therapy: Oestrogen and Lactobacilli Acidophili ovule plus pelvic floor rehabilitation (1 mg oestrogen + 50 mg lyophilisate)	Oestrogen ovule plus pelvic floor rehabilitation (1 mg oestrogen)	Capobianco et al. 2014
Oestrogen cream 0.5 mg and benzidamine	Oestrogen cream 0.5 mg	Melis et al. 1997

#### Risk of bias

**Fig 2:**
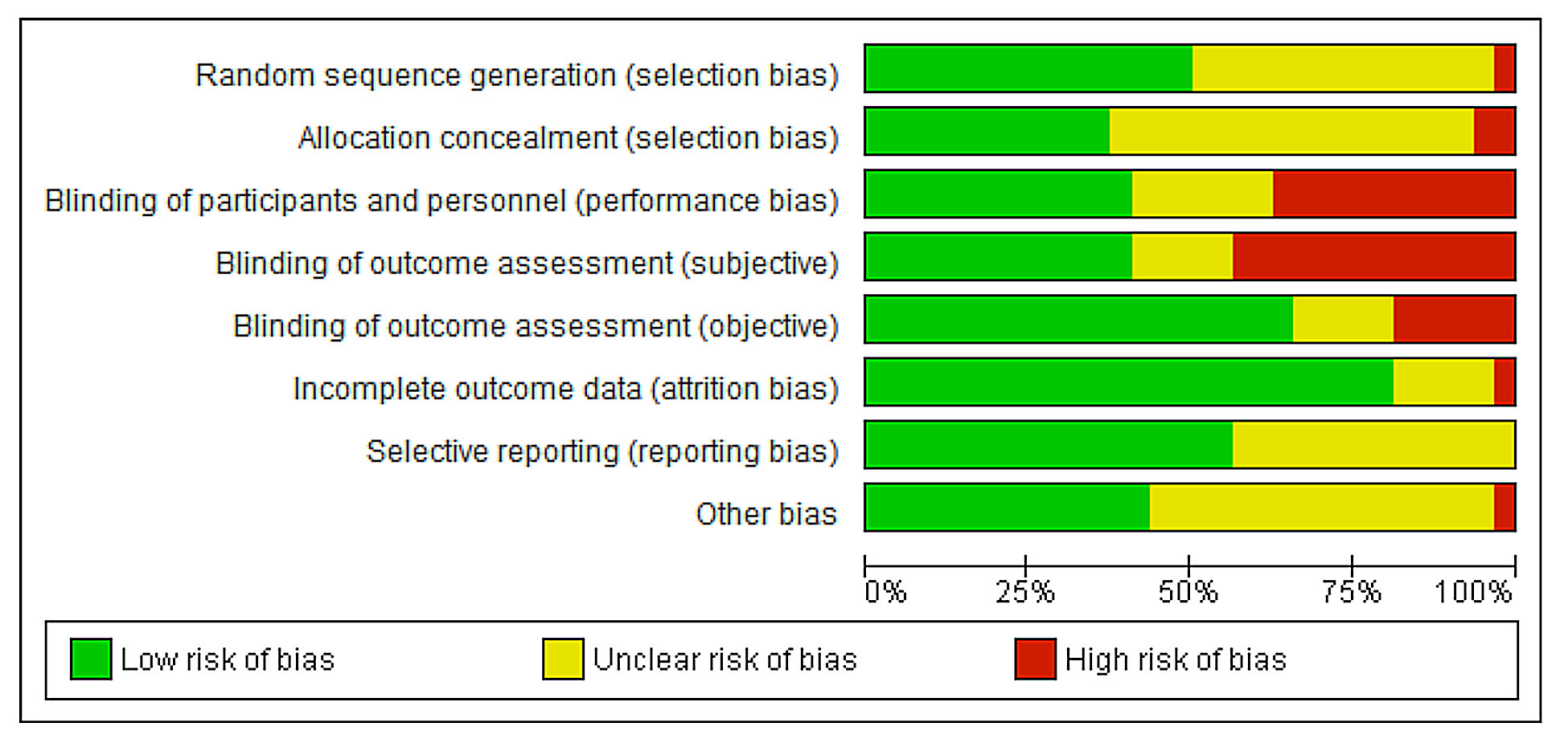
Risk of bias graph: review authors’ judgements about each risk of bias item presented as percentages across all included studies regarding local oestrogen treatment for vaginal atrophy.

**Fig 3:**
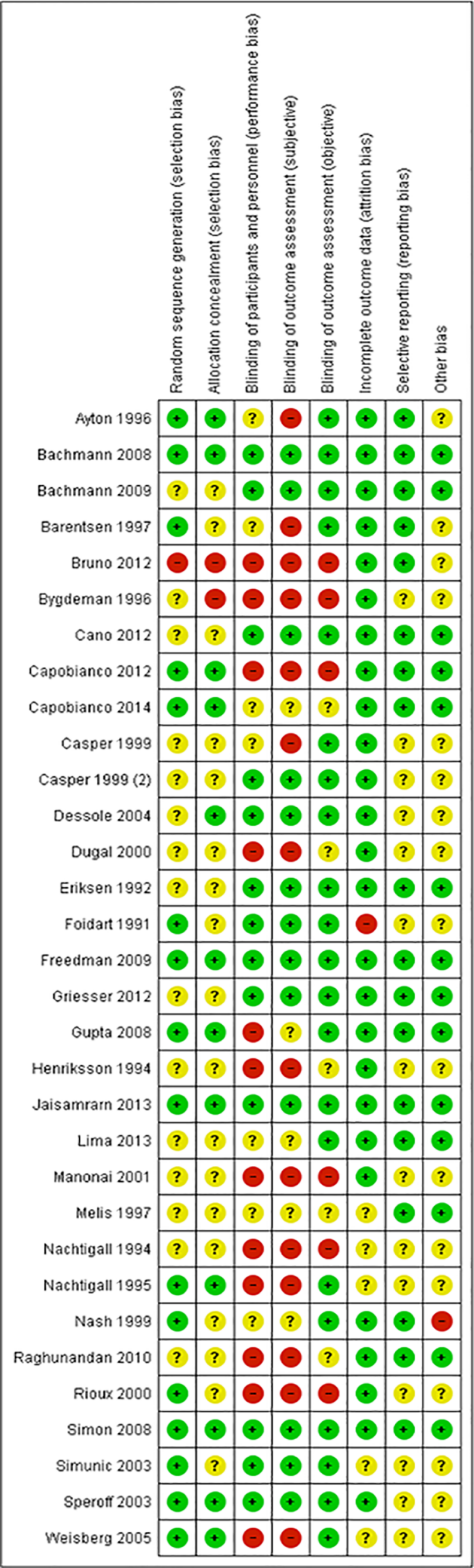
Risk of bias summary: review authors’ judgements about each risk of bias item for each included study regarding local oestrogen treatment for vaginal atrophy

As can be seen in Figs 2 and 3, about half of the trials used an adequate method of randomization (randomization sequence and allocation concealment). Fifteen trials did not report details on the randomization method [22,27,28,33,37,40,44–51]. In studies that compared different forms of applications the women could not be blinded. Blinding of the outcome assessors was often unclear. Dropouts or losses to follow up were reported in most trials included in the review and were acceptable and comparable between groups in most studies. In two studies the percentage of dropouts exceeded 20% [52,53]. The studies published after the Cochrane review all reported all predefined outcomes. In the Cochrane review reporting bias was not assessed [9]. In two studies there was a statistically significant difference in age between the treatment groups [42,54]. The risk of bias tables in the Cochrane review did not provide enough details for an assessment of other types of bias [9].

#### Comparison 1: Vaginal oestrogen versus placebo

The 15 studies that compared vaginal oestrogen with placebo varied in ways of application, types of oestrogen, doses and treatment durations. Both objective and subjective outcomes were assessed by a variety of measures. Grouping studies by outcome was therefore often not possible, resulting in analyses with three studies at the most.

After 12 to 52 weeks of treatment, women treated with local oestrogen experienced less often symptoms associated with vaginal atrophy or rated symptoms as less severe compared to the placebo treated women (analysis 1–9, S2 Appendix). The women treated with local oestrogen showed less signs of vaginal atrophy at physical examination, had an increase in vaginal maturation and had lower pH values compared to the placebo treated women (analysis 10–21, S2 Appendix). Most studies reported results after 12 weeks of treatment. Results were consistent across studies and type of oestrogen application and the differences were statistically significant. Adverse events occurred at similar rates in both the oestrogen and placebo treated groups; severe adverse events were rarely reported (analysis 22–25, S2 Appendix).

#### Comparison 2: Different application methods of vaginal oestrogen

Seven studies compared an oestrogen vaginal ring to another method of local oestrogen application (patch, tablet, cream, pessary) [36,37,41,49,53,55,56] and two studies compared an oestrogen vaginal tablet to an oestrogen cream [27,57].

When comparing different application methods of oestrogen no clear differences in symptoms, signs, pH value and adverse events could be demonstrated (analysis 26–41, S2 Appendix). The number of women per comparison was small however, resulting in wide confidence intervals. Treatment duration varied between 12 and 48 weeks; most outcomes were reported for 12 weeks of treatment.

#### Comparison 3: Vaginal oestrogen versus non-hormonal treatment

The two studies that compared vaginal oestrogen to a non-hormonal treatment both looked at the effect of vaginal oestrogen cream versus a non-hormonal vaginal gel (Replens). In both studies participants were treated for a period of 12 weeks [44,51].

Comparison between oestrogen and non-hormonal treatment (Replens) showed inconsistent results regarding vaginal pH (analysis 42, S2 Appendix) and no differences between groups regarding symptoms (data regarding symptoms could not be extracted for data analysis). Signs seemed to improve more with vaginal oestrogen treatment (analysis 43–46, S2 Appendix). Both studies described no difference in adverse events between the two groups, serious side effects were not reported.

#### Comparison 4: Different doses of vaginal oestrogen

This comparison group consisted of five trials. Bachmann and co-workers studied treatment with a vaginal oestrogen tablet containing 0.01 mg or 0.025 mg estradiol (E2) [58]. Nash and co-workers investigated the use of a vaginal ring containing E2 0.14 mg/day versus a vaginal E2 ring 0.06 mg/day [42]. Speroff evaluated treatment with a vaginal ring containing 0.05 mg/day or 0.1 mg/day [43]. Bruno assessed promestriene (Colpotrofine) 1% cream versus estriol (Ovestrion) 0.1% cream [54] en Dugal a 0.025 mg 17 β-estradiol vaginal tablet versus 0.5 mg estriol [46].

No obvious differences were reported in the comparison of different doses of vaginal oestrogen regarding symptoms of VA with the exception of two studies showing a significant decrease in vaginal dryness in favor of a higher dose of oestrogen [46,54].

Concerning signs at physical examination Bachmann and co-workers graded vaginal health with the use of a compound score of five parameters assessed over time (secretions, epithelial surface thickness and integrity, vaginal color, and pH). At week 7 the mean score for the 0.025 mg estradiol (E2) group was significantly lower (meaning less vaginal atrophy) than for the 0.01 mg E2 group. No data could be extracted for data analysis [58]. Other studies evaluating signs did not reveal significant differences between treatment groups [42,46].

The studies evaluating vaginal maturation did not show statistically significant differences between groups (analysis 47 and 48, S2 Appendix) [42,43,46,58].

Bachmann and co-workers described the percentage of participants with pH less than 5 after 12 weeks of treatment as 51% and 39% in the 0.025 mg and 0.01 mg E2 groups respectively. However it was unclear if this outcome was statistically significant [58]. The other studies in this comparison group did not evaluate vaginal pH as an outcome measure.

No differences in adverse events were reported and overall adverse events were mild to moderate (analysis 49, S2 Appendix) [43,46,54,58].

#### Comparison 5: Vaginal oestrogen versus combination therapy including vaginal oestrogen

Raghunandan and co-workers evaluated vaginal oestrogen cream and combined oestrogen and testosterone cream [22]. Capobianco and co-workers investigated in 2012 treatment with a vaginal oestrogen ovule versus a similar ovule in combination with pelvic floor rehabilitation (PFR), together called ‘combination therapy’ [32]. In 2014 they evaluated treatment with a vaginal oestrogen ovule in combination with PFR versus an oestrogen ovule in combination with PFR and Lactobacilli Acidophili (together called ‘triple therapy’) [59]. Melis and co-workers studied oestrogen cream versus oestrogen cream combined with benzidamine (an anti-inflammatory and anti-bacterial compound) [50]. Participants were treated for three to six months. Sample sizes ranged from 50–206 participants.

Combination therapy and triple therapy seemed to have more beneficial effects regarding symptoms and signs of VA, vaginal cytology and pH when compared to oestrogen alone and oestrogen plus PFR respectively (analysis 50–59, S2 Appendix).

The improvement in symptoms, signs and vaginal cytology seemed comparable between treatment with oestrogen cream and oestrogen plus testosterone cream (analysis 60–62, S2 Appendix), but the sample size was small, resulting in wide confidence intervals.

Melis and co-workers did not provide data in enough detail to include in the analysis but reported that combining oestrogen with benzidamine was significantly more effective for treatment of vaginal symptoms compared to oestrogen alone [50]. They did not describe a difference in increase in superficial cells between both groups. In all four studies no significant adverse events were reported.

### Topical oestrogens to treat urinary incontinence

#### Studies

**Fig 4:**
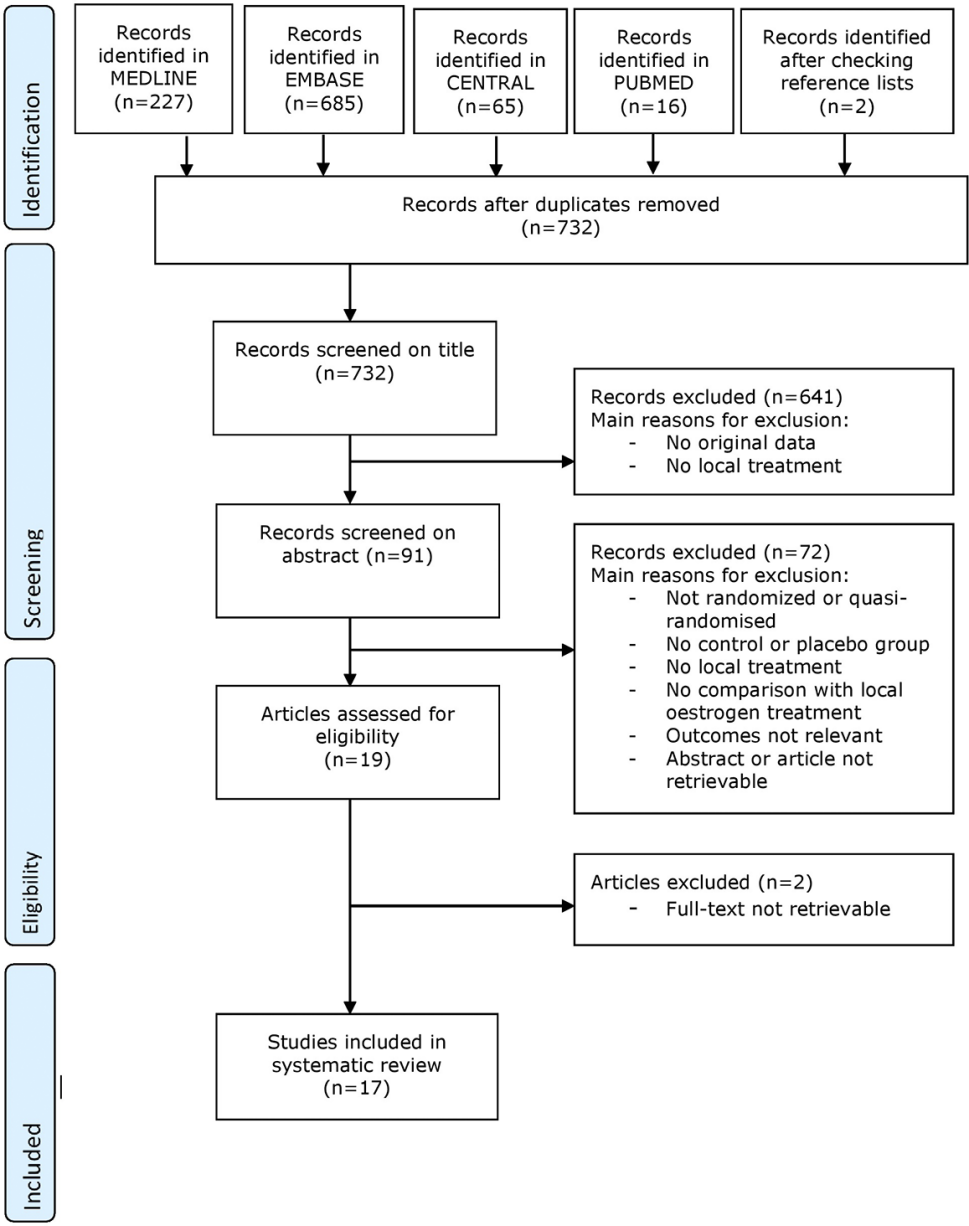
Flow chart local oestrogens for urinary incontinence and overactive bladder.

Our literature search resulted in a total of 732 studies. Main reasons for exclusion are provided in the flow chart (Fig 4). Seventeen studies were eligible for this review, of which ten were also included in the Cochrane review of Cody and co-workers [10]. Four studies were also included in the VA search [23,32,33,59].

As can be seen in Table 2, local administration of oestrogen was compared to placebo or no treatment [23,33,60–65] or non-hormonal treatment [64–69]. The remaining studies compared different application forms, doses or including another treatment [32,59,61,70–72]. The 17 studies included over 3100 participants. Treatment ranged from three weeks up to 12 months. Fourteen of the 17 studies presented their data with sufficient detail to include in the quantitative analysis.

**Table 2:** Comparisons in studies regarding local oestrogen treatment for urinary incontinence and/or overactive bladder symptoms.

Intervention	Comparison	Studies
***Vaginal oestrogen versus placebo or no treatment***		
Oestrogen 0,025–3 mg	Placebo	Simunic et al. 2003; Cardozo et al. 2001; Dessole et al. 2004; Sacco et al. 1990; Enzelsberger 1991–1; Enzelsberger 1991–2
Triple therapy (local oestrogen (dose unclear), physiotherapy and electrostimulation)	No treatment	Holtedahl et al. 1998
Premarin cream 2 gr/night (1.25 mg oestrogen)	No treatment	Henalla et al. 1989; Henalla et al. 1990
***Different application methods of vaginal oestrogen***		
Oestrogen Ring 0,5mg	Oestrogen Pessary 0,5mg	Lose et al. 2000
***Vaginal oestrogen versus non-hormonal treatment***		
Oestrogen cream 0,625mg + tolterodine	Tolterodine	Tseng et al. 2009; Serati et al. 2009
Oestrogen ring 1mg (7,5microgram/24hr)	Oral oxybutynin	Nelken et al. 2011
Oestrogen suppository 1mg	Phenylpropanolamine	Beisland et al. 1984
Premarin cream 2 gr/night (1.25 mg oestrogen)	Pelvic floor exercises	Henalla et al. 1989; Henalla et al. 1990
Premarin cream 2 gr/night (1.25 mg oestrogen)	Electrostimulation	Henalla et al. 1989
***Different doses of vaginal oestrogen***		
1mg (application form unclear)	3mg (application form unclear)	Enzelsberger 1991
0,5-1mg (application form unclear)	2mg (application form unclear)	Enzelsberger 1990
***Vaginal oestrogen versus oral oestrogen***		
Oestrogen cream 0,625mg	Oral oestrogen 0,625mg	Long et al. 2006
***Vaginal oestrogen vs combination therapy***		
Oestrogen ovule 1mg + PFR	Oestrogen Ovule 1mg	Capobianco et al. 2012
Triple therapy: Oestrogen and Lactobacilli Acidophili ovule plus pelvic floor rehabilitation (1mg oestrogen + 50 mg lyophilisate)	Oestrogen ovule plus pelvic floor rehabilitation (1mg oestrogen)	Capobianco et al. 2014

#### Risk of Bias

**Fig 5:**
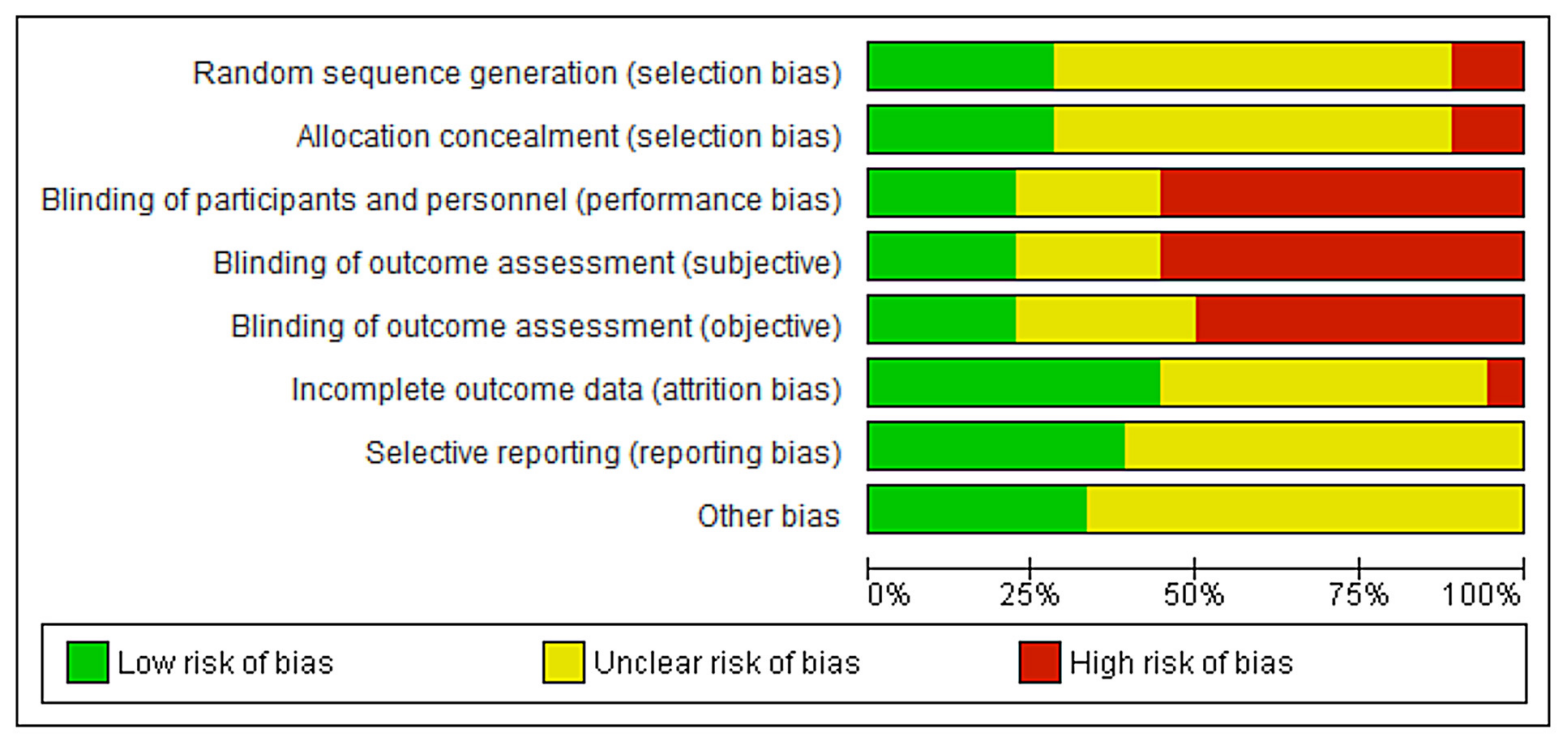
Risk of bias graph: review authors’ judgements about each risk of bias item presented as percentages across all included studies regarding local oestrogen treatment for urinary incontinence and/or overactive bladder.

**Fig 6:**
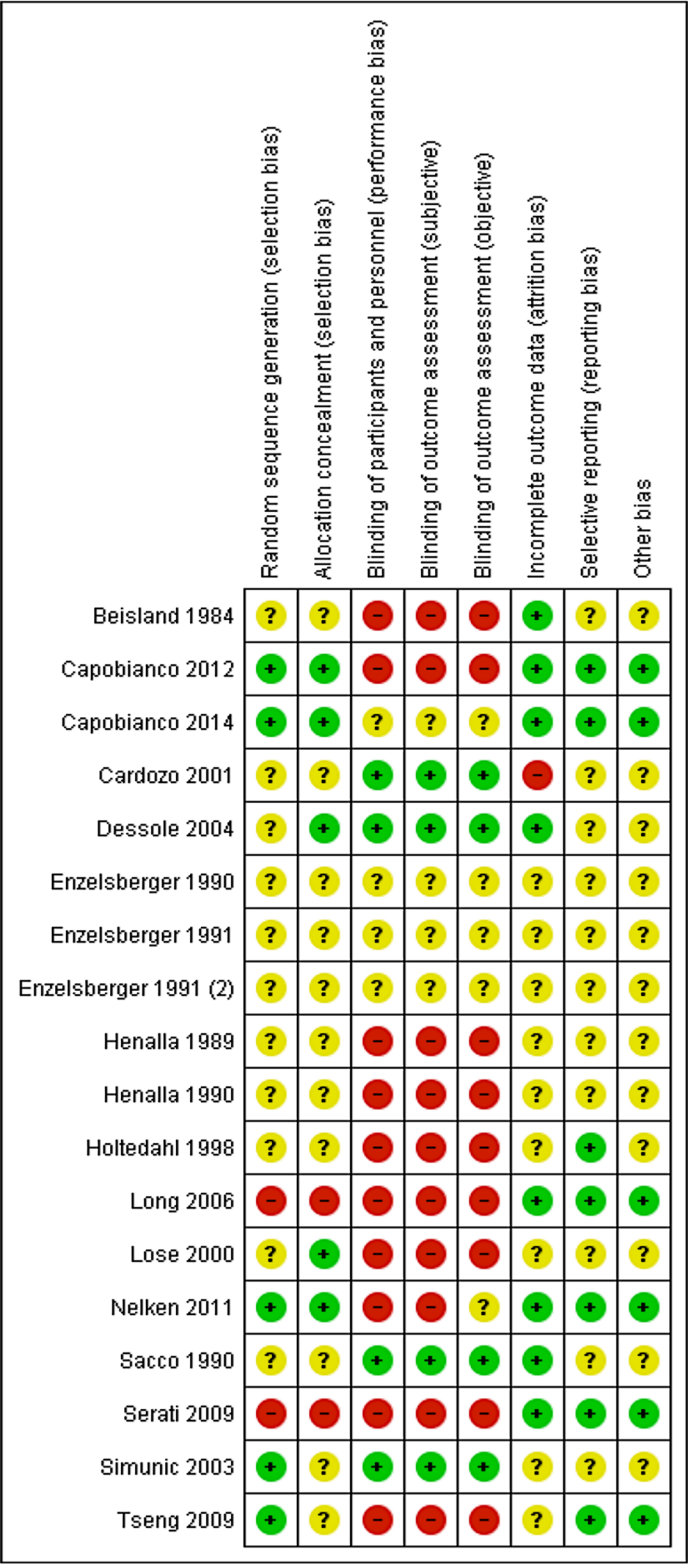
Risk of bias summary: review authors’ judgements about each risk of bias item for each included study regarding local oestrogen treatment for urinary incontinence and/or overactive bladder.

As can be seen in Figs 5 and 6, five studies used an adequate method of randomization [23,32,59,67,69]. Two studies had high risk of selection bias [68,72] and in ten studies it was unclear whether an adequate random sequence generation method was used [33,60–66,70,71]. Almost half of the studies were not blinded, and in four studies blinding was unclear. Four studies were double blinded [23,33,60,62]. Dropouts or losses to follow up were reported in most of the trials, with acceptable dropout rates. Studies included after the search date of the Cochrane review were at low risk of reporting bias. In one study we found baseline differences [63]. The risk of bias assessment of the studies included in the Cochrane review did not include reporting bias or details on other biases [10].

#### Comparison 1: Vaginal oestrogen versus placebo or no treatment

This group consisted of eight studies of which five studies used a placebo controlled design [23,33,60–62], two studies compared vaginal oestrogen to no treatment [64,65] and one study compared vaginal oestrogen (in a combination therapy with physiotherapy and electrostimulation) with a no treatment group [63].

Two studies applied vaginal oestrogen with the use of ovules [33,63], two studies used oestrogen vaginal tablets [23,60] and three studies prescribed an oestrogen vaginal cream [62,64,65]. In one study the method of application was unclear [61].

Overall, subjective, semi-objective and urodynamic outcomes changed in favor of the vaginal oestrogen groups compared to placebo (analysis 1–4, S3 Appendix).

Treatment side effects were equally distributed in the vaginal oestrogen and placebo group in the study of Simunic and co-workers. No severe adverse events were reported [23]. Three other studies reported no significant differences in adverse events between groups [33,60,61].

#### Comparison 2: Different application methods of vaginal oestrogen

One study compared a vaginal oestrogen ring with a vaginal oestrogen pessary in a group of 251 postmenopausal women for a treatment period of 24 weeks [70].

Response to treatment with respect to stress and urge incontinence, urgency, frequency, dysuria and nocturia was comparable between groups. The overall subjective judgment (acceptability) of the type of administration was in favor of the vaginal ring (analysis 5, S3 Appendix), this was not based on an experience with both application methods. The percentage of women reporting side effects in the pessary group was slightly higher compared to the women in the ring group (35.9% versus 25.4%), but the difference was not statistically different (analysis 6, S3 Appendix).

#### Comparison 3: Vaginal oestrogen versus non-hormonal treatment or combination treatment

Two studies compared vaginal oestrogen cream combined with tolterodine (a competitive cholinergic receptor antagonist) with tolterodine alone [68,69]. One study evaluated the differences between treatment with an oestrogen vaginal ring and oral treatment with oxybutynin (an anticholinergic drug used for relaxation of the detrusor muscle) [67]. Another study compared a vaginal oestrogen suppository to phenylpropanolamine (PPA; a nonselective adrenergic receptor agonist and norepinephrine reuptake inhibitor) [66]. Two studies assessed Premarin vaginal cream versus PFR [64,65] and one of these studies also evaluated Premarin vaginal cream versus pelvic floor electrostimulation (interferential therapy) [65].

Treatment effects with vaginal oestrogen only or non-hormonal treatment (combined or not combined with vaginal oestrogen) were largely similar regarding subjective efficacy (analysis 7–11, S3 Appendix).

Bladder diary variables improved slightly more in the tolterodine plus oestrogen group as compared to the tolterodine alone group in a small study of 40 participants in each group (analysis 12 and 13, S3 Appendix); the differences were however, not statistically significant for most outcomes [69]. In another study, users of an estradiol vaginal ring and users of oral oxybutinine both reported a lower frequency of daily voids after 12 weeks treatment, but these decreases were not statistically significant (analysis 14, S3 Appendix) [67]. In two small studies the percentage of women with stress urinary incontinence that were cured or improved was lower in the oestrogen group compared to the groups with pelvic floor exercises or electrostimulation, For the comparison with exercise this difference was statistically significant (analysis 15 and 16, S3 Appendix) [64,65].

Increase in MUCP seemed greatest in patients treated with oestrogens compared to pelvic floor exercises or electrostimulation [65]. No statistically significant difference in urodynamic variables were reported between PPA and estriol when administered separately [66] (data could not be extracted for analysis).

Vaginal discharge occurred more often in the ring groups while complaints of dry mouth and constipation occurred more in the oxybutynin group (analysis 17, S3 Appendix) [67]. Tseng and co-workers described no significant adverse events in both treatment groups (tolterodine/oestrogen vs tolterodine alone, 80 women in total) [69]. Beisland and co-workers (n = 20) reported one patient with genital bleeding during combined treatment (PPA/oestrogen) and one patient with insomnia after three days of PPA treatment [66].

#### Comparison 4: Different doses of vaginal oestrogen

Two studies, both performed by Enzelsberger and co-workers, assessed the differences in symptoms of urge urinary incontinence between treatment with different doses of vaginal oestrogen [61,71]. Both studies were unclear about the method of application, only mentioning the different doses in both treatment groups. In the first study, 0.5–1 mg vaginal oestrogen was compared to 2 mg vaginal oestrogen [71]. The other study compared 1 mg vaginal oestrogen to 3 mg vaginal oestrogen (and placebo, see analysis 2 and 3, S3 Appendix) [61].

Regarding bladder diary variables data from the 0.5–1 mg group in the study of Enzelsberger and co-workers in 1990 seemed to be the same as the data from the 1 mg group in their study in 1991 (analysis 18 and 19, S3 Appendix). In the first study, nocturia as well as micturition frequency decreased significantly more compared to baseline in the high dose group compared to the low dose group [71]. In the second study, nocturia occurred significantly less frequent after treatment in the high dose group (3 mg). The difference in urinary frequency between the two groups was not significant [61].

No significant differences regarding urodynamic variables between high dose and low dose treatment groups in both studies were found except for the difference in bladder capacity; the results suggest that the capacity increased with increasing dose. However confidence intervals were large (analysis 20 and 21, S3 Appendix).

#### Comparison 5: Vaginal oestrogen versus oral oestrogen

One study investigating vaginal versus oral oestrogen therapy was included. This study compared treatment with oestrogen vaginal cream to oral oestrogen in the same dose in 57 postmenopausal women [72].

Oral oestrogen seemed to be more effective in decreasing urinary frequency compared to vaginal cream whereas vaginal cream was more effective in decreasing nocturia. Changes of other symptoms, including stress and urge urinary incontinence, were not statistically significantly different between the two groups (analysis 22, S3 Appendix). Among improved patients there was no statistically significant difference in the mean number of stress urinary incontinence episodes per week (analysis 23, S3 Appendix). Adverse events were not reported.

#### Comparison 6: Vaginal oestrogen versus combination therapy including vaginal oestrogen

Two studies provided evidence for the effectiveness of combination therapy. Compared to oestrogen therapy alone, combining PFR with oestrogen resulted in higher mean values for all urodynamic outcomes (statistically significant except for bladder capacity) [32]. In the second study, Lactobacilli acidophilli was added to treatment with PFR and oestrogen. Compared to oestrogen therapy plus PFR, the mean values of all urodynamic outcomes were higher in the triple therapy group with statistically significant differences for MUP, MUCP and the pressure transmission ratio (PTR) [59] (analysis 24–27, S3 Appendix).

In both studies no systemic adverse reactions were observed.

### Topical oestrogens to treat pelvic organ prolapse

#### Studies

**Fig 7:**
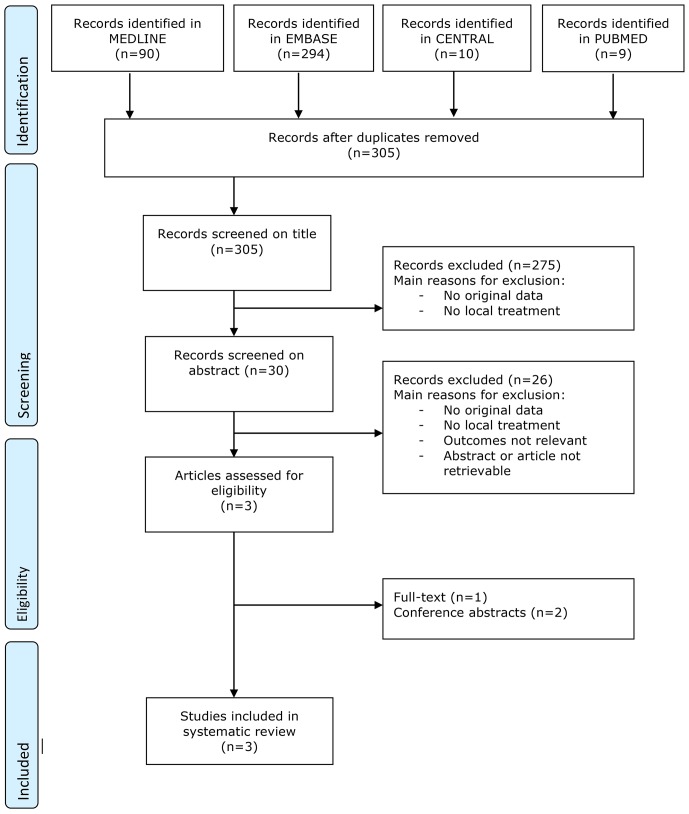
Flowchart local oestrogens for pelvic organ prolapse.

Our literature search on local oestrogen therapy for pelvic organ prolapse resulted in a total of 305 studies (Fig 7). Main reasons for exclusion were the absence of original data of local treatment, irrelevance of outcomes or irretrievable abstracts. This left us with three studies, of which one was available in full text [73]. For the other two studies we used the abstracts to extract information from [74,75]. One study was also included in the Cochrane review of Ismail and co-workers, published in 2010 [19,73].

As can be seen in Table 3, two out of the three studies investigated local administration of oestrogen versus placebo or no treatment [73,75]. One study compared local oestrogen therapy in combination with oral duloxetine (a selective serotonin and norepinephrine reuptake inhibitor) and Kegel exercises to surgical treatment (anterior colporrhaphy) [74]. In the studies 16 to 48 women were enrolled. The duration of treatment ranged from two weeks up to three months. None of the studies reported data that could be included in the quantitative analysis.

**Table 3:** Comparisons in studies regarding local oestrogen treatment for pelvic organ prolapse.

Intervention	Comparison	Studies
***Vaginal oestrogen versus placebo or no treatment***		
Oestrogen Pessary 25 microgram	Placebo	Felding et al. 1992
Oestrogen Cream 1 gram (dose of oestrogen unclear)	No treatment	Vaccaro et al. 2011
***Vaginal oestrogen versus surgical treatment***		
Vaginal oestrogen 25 microgram (+ duloxetine p.o. + Kegel exercises)	Anterior colporrhaphy	Nikas et al. 2012

#### Risk of bias

**Fig 8:**
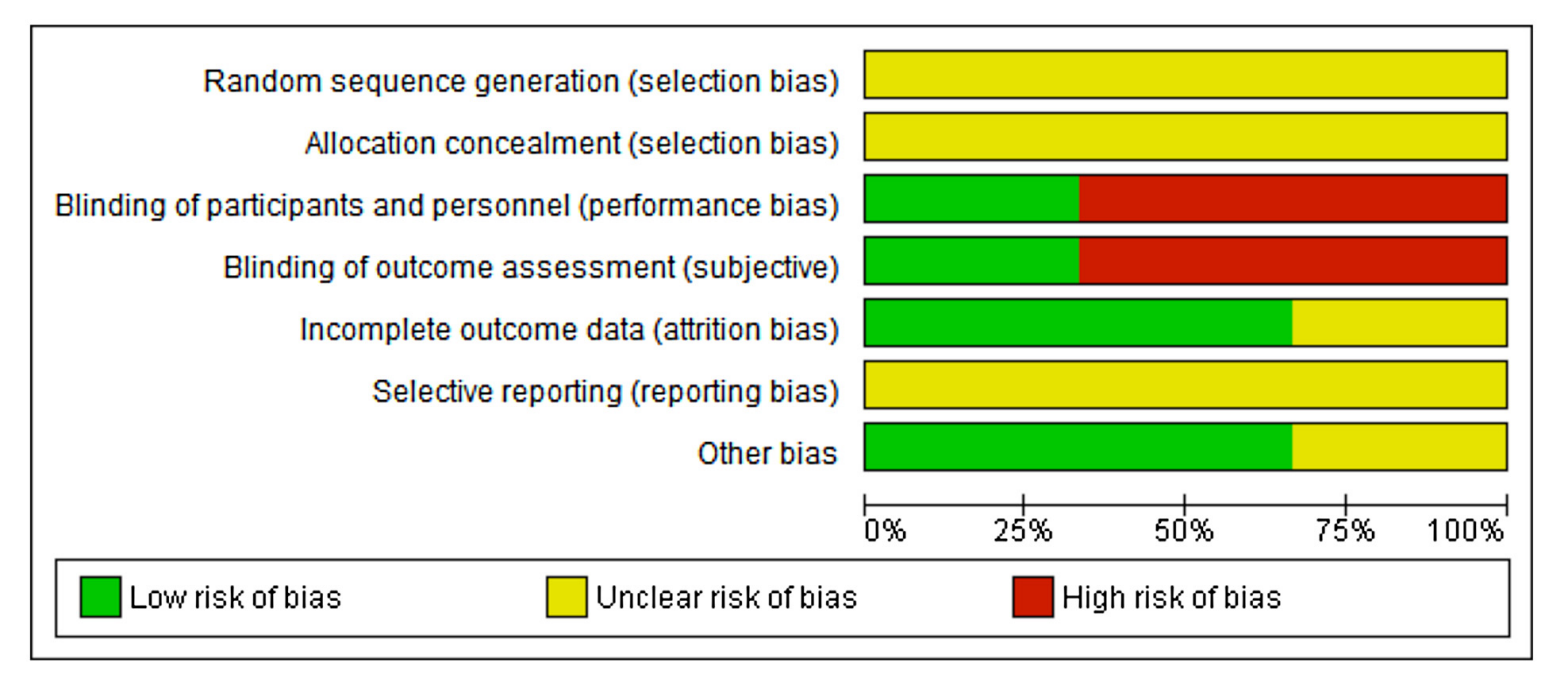
Risk of bias graph: review authors’ judgements about each risk of bias item presented as percentages across all included studies regarding local oestrogen treatment for pelvic organ prolapse.

**Fig 9:**
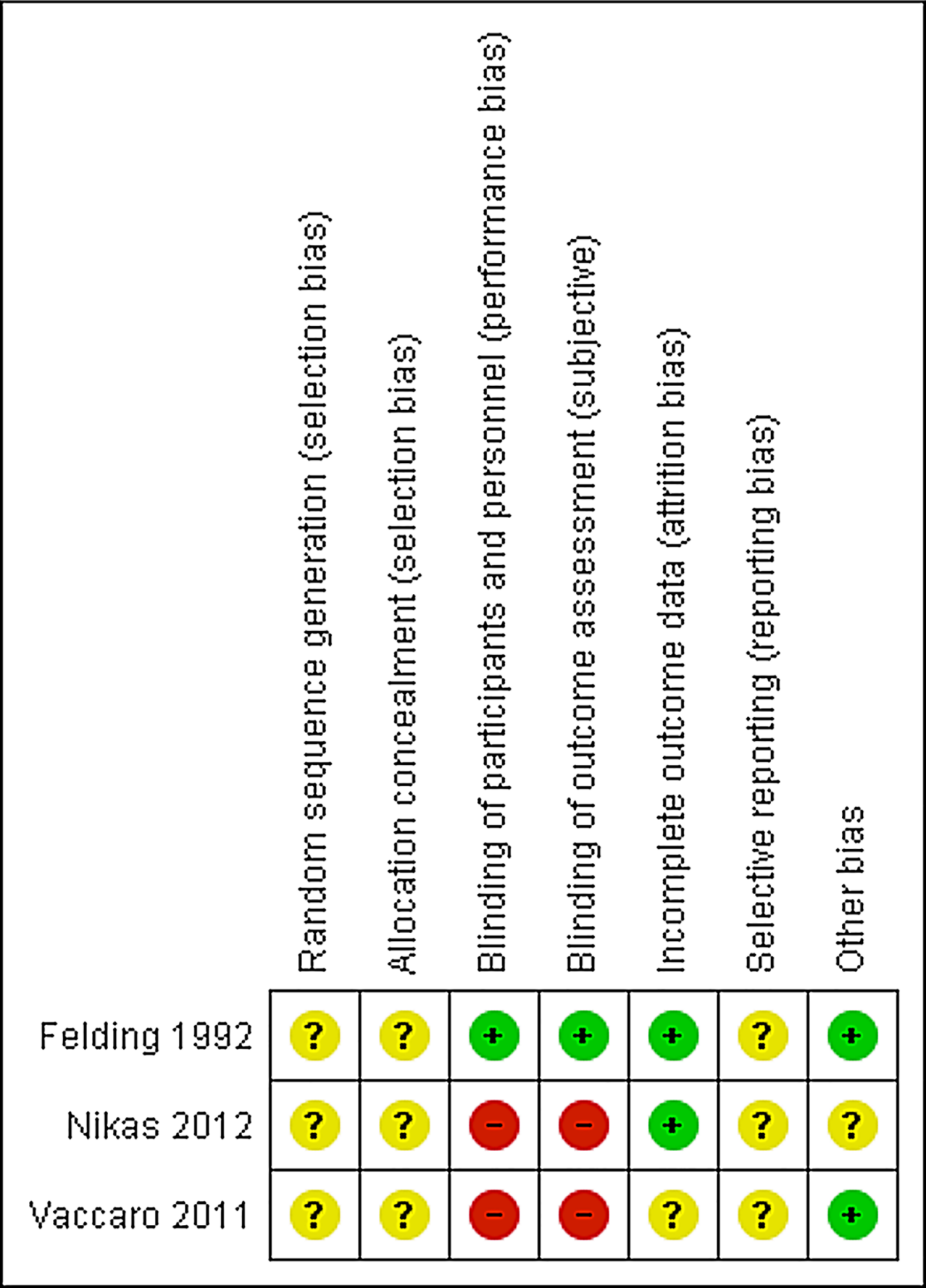
Risk of bias summary: review authors’ judgements about each risk of bias item for each included study regarding local oestrogen treatment for pelvic organ prolapse.

As can be seen in Figs 8 and 9, all three studies were unclear about the randomization method. One study had a double blind design [73]; the other studies were not blinded. Two studies reported acceptable rates of dropout or no dropouts. One study enrolled 40 women of which 70% had completed treatment at the time of publication of the abstract [75]. From all three studies it was not clear if all predefined outcomes were reported. In two studies there were no differences in baseline characteristics between the different treatment groups [73,75]. In one study insufficient information was provided [74].

#### Comparison 1: Vaginal oestrogen versus placebo or no treatment

One study compared treatment with a vaginal oestrogen pessary to placebo [73]. The other study compared vaginal oestrogen cream to no treatment [75].

Vaccaro and co-workers described a statistically significant difference in vaginal symptoms (dryness, soreness, and irritation) on a visual analogue scale (VAS) between the treatment group and no treatment group in favor of the treatment group [75]. Felding and co-workers did not describe (differences in) decrease of relevant symptoms [73].

Felding and co-workers reported that thickness of the vaginal wall (assessed histologically with the use of a vaginal wall punch biopsy) was statistically significantly more increased in the treatment group compared to the placebo group [73]. Vaccaro and co-workers described a statistically significant difference in Vaginal Health Composite Score (VHCS; evaluates the amount of epithelial rugosity/integrity, vaginal color, pH and secretions) between the treatment group and no treatment group in favor of the treatment group [75]. No adverse events were reported.

#### Comparison 2: Vaginal oestrogen versus surgical treatment

One study evaluated the efficacy of treatment with 25 microgram estradiol vaginally plus 40 mg of Duloxetine orally and Kegel exercises versus anterior colporrhaphy in 16 women with stage I cystocele [74].

All women reported less vaginal dryness, no information was provided regarding POP symptoms. No case of progress of the cystocele was reported during oestrogen treatment. Post-operative examination in the group that underwent anterior colporrhaphy revealed successful treatment of the cystocele.

## Discussion

This is a review providing a systematic, extensive overview of the effects of local oestrogen treatment on pelvic floor disorders including vaginal atrophy, urinary incontinence, overactive bladder symptoms as well as pelvic organ prolapse. We intended to perform a meta-analysis of the available data. However, due to wide variation in outcome assessment of different pelvic floor symptoms, the variation in type and dosage of the investigated oestrogen treatment regimens and the variety in comparisons made, it was not feasible to perform a proper meta-analysis. As a result this systematic review mainly summarizes and discusses the outcome and interpretation of individual studies. We investigated the risk of bias of the included studies to ensure the integrity of conclusions drawn regarding subjective and objective efficacy and safety. Before further interpreting these conclusions potential limitations need to be addressed.

Although we systematically and thoroughly reviewed the available literature, results need to be interpreted cautiously due to small sample sizes in most studies, variation in trial design, wide variation in outcomes assessing efficacy and safety and a variation in type and dosage of oestrogen used. Moreover, because of the many different definitions of VA, combining study results was often not possible. These limitations in the data analysis resulted in imprecise effect estimates [8]. Additionally, data for each of the pre-defined outcome measures of this review were not available in all trials and the analysis of the outcome measures could only be based on the studies where data extraction was possible, which raised the probability of selective reporting. However, we compared the conclusions of the studies for which data extraction was not possible with the studies included in the analysis, and these were in line. The risk of bias of the included studies was generally moderate, mainly caused by inadequate blinding of participants and personnel (performance bias).

We did not evaluate serum absorption or endometrium stimulation of the different topical oestrogens in the assessment of safety. Low dose vaginal oestrogens have been used for many years in the treatment of women with pelvic floor disorders and recently published guidelines have shown them to be safe [76]. For this review we were more interested in differences in local adverse events between the treatments examined which could cause discontinuation of treatment (i.e. vaginal discharge, irritation).

Tolerability of the different application methods of topical oestrogen was not examined. Suckling and co-workers previously stressed the difficulty of comparing participant acceptability between the ring, cream and tablets due to the differences in frequency of administration. They did suggest a better tolerability of the vaginal ring over other topical vaginal oestrogen preparations due to its delivery system and ease of use. However, in the studies that led to this conclusion, women with pelvic organ prolapse were often excluded from participation. The ring may not be suitable to those with limited vaginal space or pelvic organ prolapse [77]. For that reason it cannot be concluded which application method is accepted best by the patient.

We did not specifically differentiate between different types of oestrogen (i.e. estradiol, estriol, promestriene, synthethic conjugated estrogens) in the description of our results. This subdivision has been previously made by Rees and co-workers in the EMAS clinical guide published in 2012 [76]. With this review we aimed to assess if oestrogens in general are effective in different pelvic floor disorders.

### Summary of findings regarding local oestrogen treatment for vaginal atrophy

Studies comparing vaginal oestrogen treatment to placebo were consistent in reporting more beneficial effects on symptoms and signs of VA, vaginal maturation and vaginal pH after oestrogen treatment. Regarding most subjective and objective outcome measures no obvious differences between the different application methods were found. Comparison between oestrogen and non-hormonal treatment (Replens) showed no differences in outcome as far as symptoms and vaginal pH. Signs at physical examination seemed to improve more with vaginal oestrogen treatment [44,51]. No obvious differences were identified in the comparison of different doses of vaginal oestrogen with the exception of two studies showing a significant decrease in vaginal dryness in favor of a higher dose of oestrogen [46,54]. Combination therapy (vaginal oestrogen ovule combined with PFR and/or Lactobacilli Acidophili) seemed to have more beneficial effects regarding symptoms and signs of VA, vaginal cytology and pH as compared to only oestrogens [32,59].

### Summary of findings regarding local oestrogen treatment for urinary incontinence and overactive bladder

Overall, subjective, semi-objective and urodynamic variables changed in favor of the vaginal oestrogen groups compared to placebo. No obvious differences in efficacy outcome measures between different application methods were revealed. Treatment with vaginal oestrogen only or non-hormonal treatment (combined or not combined with vaginal oestrogen) was similar regarding subjective efficacy. In two small studies there was a significant reduction in pad weights in the group that received pelvic floor exercises compared to oestrogen treatment [64,65]. Increase in MUCP seemed greatest in patients treated with oestrogens compared to pelvic floor exercises or electrostimulation [65]. Bladder diary variables improved in favor of the tolterodine plus oestrogen group compared to the tolterodine only group in a small study of 40 participants in each group [69]. Nocturia seemed to decrease more after treatment in a high dose (2 mg or 3 mg) group compared to a low dose group (0.5–1 mg) [61,71]. Oral oestrogen seemed to be more effective in decreasing urinary frequency compared to vaginal cream whereas vaginal cream was more effective in decreasing nocturia [72]. Combination therapy (vaginal oestrogen ovule combined with PFR) and triple therapy (oestrogen ovule in combination with PFR and Lactobacilli Acidophili) caused significantly more improvement in symptoms of stress urinary incontinence [32,59].

### Summary of findings regarding local oestrogen treatment for pelvic organ prolapse

The studies evaluating the effect of local oestrogen treatment versus placebo or no treatment in women with POP mainly assessed symptoms and signs associated with VA instead of POP symptoms (i.e. sense of pressure or bulge vaginally) [73,75]. This was the same in the study comparing conservative treatment with 25 microgram estradiol vaginally plus 40 mg of Duloxetine orally and Kegel exercises versus anterior colporrhaphy [74]. With these findings and the lack of studies investigating the effect of vaginal oestrogen treatment on POP symptoms, the potential for local oestrogens in the prevention as well as treatment of POP needs to be further established.

### Implications for research

Our findings are consistent with the Cochrane reviews regarding local oestrogen treatment for vaginal atrophy published in 2006 [9], for urinary incontinence published in 2012 [10] and for POP published in 2010 [19].

Suckling and co-workers recommended in 2006 that intra-vaginal oestrogenic preparations versus placebo should be researched more. Since their review, an additional 10 placebo-controlled studies have been published and these were included in our review. They also recommended that additional trials providing long-term data (over six months) about efficacy and safety were required. Unfortunately, such long-term studies are still lacking.

With respect to OAB symptoms it was already described by Robinson and co-workers that there is some evidence that vaginal oestrogen could be useful in the management of OAB symptoms [15]. We included two studies evaluating the effect of local oestrogen treatment on overactive bladder symptoms that were excluded in the Cochrane review of 2012 because not all participants had urinary incontinence at baseline. Long and co-workers described that local oestrogen could relieve OAB symptoms while Serati and co-workers described no synergistic effect of local oestrogen when administered together with antimuscarinic medication [68,72]. Bladder diary variables improved slightly more when tolterodine was combined with topical oestrogen compared to tolterodine only in a small study of 40 participants [69].

Regarding local oestrogen treatment for pelvic organ prolapse further research should focus on the effect of oestrogen treatment on POP symptoms and signs. There is a need for well-organized randomized controlled trials with adequate sample size comparing topical oestrogen treatment to placebo evaluating at least symptoms of sense of pressure or bulge vaginally, self-reported improvement or cure of symptoms, quality of life related to pelvic floor symptoms, delay or no need for alternative treatments and clinicians observations of improvement of POP using the POP-Q system.

### Implications for practice

Vaginal oestrogen treatment in the form of creams, pessaries, tablets and rings have proven to be effective and safe in the treatment of VA related symptoms at dosages of 10 mcg and more [52,58]. The differences in efficacy between different application methods are very limited. In clinical practice patient preference should guide the selection of the application method. In case pelvic organ prolapse is present, or the vagina is short, a vaginal ring is probably not the best option.

There is evidence implicating a beneficial effect of vaginal oestrogen treatment on urinary incontinence and overactive bladder symptoms, potentially combined with other treatment modalities like PFR or antimuscarinic medication. Again, the way oestrogens are administered plays a minor role. There is too little evidence to recommend on a preferred dose to realize the best outcome.

Regarding vaginal oestrogen treatment for POP the available literature is insufficient to provide evidence based recommendations for clinical practice. One can imagine that relieving VA-related symptoms in patients with POP could relief the sense of vaginal bulge associated with POP, however there are so far no robust data to support this.

### Conclusion

Pelvic floor symptoms are complex and multi-factorial. The decline in available oestrogen after menopause is a risk factor for development of worsening of pelvic floor symptoms. Topical oestrogen administration has proven to be effective for the treatment of vaginal atrophy and seems to decrease symptoms of overactive bladder and urinary incontinence. Literature suggests benefit for women with POP although more evidence is needed. Physicians treating women with pelvic floor symptoms should be aware of the capacity of topical oestrogen treatment and include it in their counseling when discussing treatment options.

## Supporting Information

S1 PRISMA Checklist PRISMA Checklist. (DOC)

S1 Appendix MEDLINE search. (DOC)

S2 Appendix Analysis local oestrogen for vaginal atrophy. (DOC)

S3 Appendix Analysis local oestrogen for urinary incontinence and OAB. (DOC)
