# Supplementary material for: Local Oestrogen for Pelvic Floor Disorders: A Systematic Review
Source: PLoS One. 2015 Sep 18;10(9):e0136265. doi: 10.1371/journal.pone.0136265 (PMC4575150; doi:10.1371/journal.pone.0136265)
Supplement: S1 Appendix — (DOC) [file pone.0136265.s002.doc]

**APPENDIX 1: MEDLINE search**

Database(s): **Ovid MEDLINE(R) In-Process & Other Non-Indexed Citations and Ovid MEDLINE(R)** 1946 to Present Search Strategy: **2014-05-25**

| **#** | **Searches** | **Results** |
| --- | --- | --- |
| 1 | (Estrace or Estring or Femring or Menoring or Promestriene or Oestro-gyn*dron or Oestrogyn*dron or Oekolp or Ortho-Gynest or Orthogynest or Ovestin or VAGIFEM or Xapro or EstroGel or EVT or GynoFlor).tw,ot,kw. | 659 |
| 2 | ((vaginal* or local* or topical*) adj3 (estrogen* or oestrogen* or estradiol* or oestradiol* or estriol* or oestriol* or estetrol* or oestetrol* or estrone* or oestrone* or dien?estrol or hydroxyestriol or Ovocyclin or Premarin or Synapause or SCE-A)).tw,ot. | 2617 |
| 3 | or/1-2 [ESTR. A] | 3224 |
| 4 | estrogens/ or estradiol/ or "estrogens, conjugated (usp)"/ or "estrogens, esterified (usp)"/ or estrone/ or ethinyl estradiol/ or estrogens, non-steroidal/ or exp estriol/ or exp Estradiol Congeners/ | 134201 |
| 5 | Estrogen Replacement Therapy/ | 13854 |
| 6 | (estrogen* or oestrogen* or estradiol* or oestradiol* or estriol* or oestriol* or estetrol* or oestetrol* or estrone* or oestrone* or dien?estrol or hydroxyestriol or Ovocyclin or Premarin or Synapause or SCE-A).tw,ot,kw. | 179468 |
| 7 | or/4-6 | 217394 |
| 8 | Administration, Intravaginal/ | 3621 |
| 9 | (intravagina* or intra-vagina*).tw,ot,kw. | 4414 |
| 10 | administration, topical/ | 32093 |
| 11 | ointments/ or suppositories/ or tablets/ or "vaginal creams, foams, and jellies"/ or gels/ or pessaries/ | 55595 |
| 12 | (ring or rings or (vaginal adj3 (tablet* or depot* or deposit* or insert or inserts or applicator*)) or vagitor* or ovule* or suppositor* or cream* or pessar* or gel or gels).tw,ot,kw. | 431554 |
| 13 | (vaginal ring* or vaginal tablet* or vagitor* or vaginal depot* or vaginal insert* or vaginal cream* or vaginal pessar*).kw. | 201 |
| 14 | ((vaginal* or local* or topical*) adj3 (adminis* or treat* or therap* or applicat* or applied or delivered)).tw,ot. | 94310 |
| 15 | or/8-14 | 570735 |
| 16 | 7 and 15 [ESTR. B] | 7864 |
| 17 | 3 or 16 [ESTR] | 10230 |
| 18 | (animals/ not humans/) or (rat or rats or mouse or mice or rodent*).ti. | 4023592 |
| 19 | 17 not 18 [ESTR human] | 6870 |
| 20 | Atrophic Vaginitis/ | 9 |
| 21 | (atroph* adj10 (vagin* or vulv* or urogenital* or genital* or genito*)).tw,ot. | 1178 |
| 22 | (d?stroph* adj3 (vagin* or vulv* or urogenital* or genital* or genito*)).tw,ot. | 183 |
| 23 | (vagin* atroph* or vulv* atroph* or atroph* vagin* or atroph* vulv*).kw. | 24 |
| 24 | (urovagi* or uro-vagi*).mp. | 41 |
| 25 | ((vagin* or vulv*) adj3 dry*).tw,ot. | 743 |
| 26 | (dry vagina or vaginal dryness).kw. | 5 |
| 27 | xeros*.tw,ot,kw. | 4099 |
| 28 | ((urogenit* or uro-genit* or genito*) adj3 (signs or symptoms or scor*)).tw,ot. | 717 |
| 29 | ((vagina* or vulva* or vulvovag*) adj2 (complaint* or discomfort* or itch* or burn* or sore or soreness or irritat* or erosion* or symptom* or signs or health or function or condition*)).tw,ot. | 2529 |
| 30 | ((urogenital* or genital* or vagina* or vulvovag* or reproductive or sexual) adj3 aging).tw,ot. | 782 |
| 31 | (bothersome adj4 symptom*).tw,ot. | 641 |
| 32 | dyspareunia/ | 1417 |
| 33 | d?spareunia.tw,ot,kw. | 2516 |
| 34 | (pain adj3 (intercours* or sex* or coitus or vagina* or vulv*)).tw,ot. | 2628 |
| 35 | (Sexual Functioning Ind* or Menopause Rating Scal* or female sexual d?sfunct* or female sexuality questionn*).tw,ot,kw. | 1034 |
| 36 | (vagina* adj3 (pH or acidity)).tw,ot. | 691 |
| 37 | vagin* pH.kw. | 2 |
| 38 | ((maturat* or maturity) adj3 (vagina* or vulvo*)).tw,ot. | 190 |
| 39 | ((maturation or maturity or karyopy?notic) adj2 (indices or index)).tw,ot. | 722 |
| 40 | (matur* ind* or karyopy?notic ind*).kw. | 12 |
| 41 | (VVA or VMI or VVSs or (VHI not voice handicap) or VHIS or PFSF or VMV or VIVA or GHCE or FSFI or (MBS not Bishop score*) or UGAQoL or UAQ or MFSQ or FSD).tw,kw. | 3605 |
| 42 | or/20-41 **[VA-1]** | 20163 |
| 43 | genitalia, female/ or vagina/ or vulva/ or vaginal diseases/ or exp vaginitis/ or vulvovaginitis/ or vulvar diseases/ or vulvitis/ or urogenital system/ | 54577 |
| 44 | Coitus/ | 6179 |
| 45 | (coitus or intercourse).tw,ot,kw. | 16967 |
| 46 | or/43-45 | 73387 |
| 47 | (atroph* or d?stroph*).mp. | 152180 |
| 48 | 46 and 47 **[VA-2]** | 1330 |
| 49 | 42 or 48 **[VA]** | 20744 |
| 50 | 49 and 19 **[VA ESTR]** | 648 |
| 51 | (meta-analysis.pt. or (meta analy* or metaanaly* or meta?analy* or ((review* or search* or research) adj10 evidence) or ((review* or search* or research or evidence) adj10 (literature* or medical database* or systemat* or exhaustive)) or medline or pubmed or embase or cochrane or cinahl or psychinfo or psychlit or healthstar or biosis or current conten*).tw,ot,kw. or cochrane.jw.) not (comment or editorial or historical-article).pt. | 416445 |
| **52** | **50 and 51 [VA ESTR SR]** | **60** |
| 53 | (randomized controlled trial or controlled clinical trial).pt,kw. or random allocation/ or double-blind method/ or single-blind method/ or (groups or subgroup*).ab. or (randomi?ed or randomly or placebo* or trial or allocat* or ((random* or controlled) adj2 study) or ((singl* or doubl* or treb* or tripl*) adj (blind*3 or mask*3))).tw,ot. | 2179560 |
| **54** | **50 and 53 [VA ESTR RCT]** | **251** |
| 55 | exp Urinary Incontinence/ | 26038 |
| 56 | urinary bladder, overactive/ | 2372 |
| 57 | lower urinary tract symptoms/ | 623 |
| 58 | nocturia/ | 385 |
| 59 | urination disorders/ | 10245 |
| 60 | Urodynamics/ | 12998 |
| 61 | Urination/ | 8206 |
| 62 | ((urin* or urolog* or bladder or stress or urge* or intercourse or coital or frequency or mixed) adj8 (incontinen* or continence)).tw,ot. | 26073 |
| 63 | ((incontinen* or continence) adj3 episode*).tw,ot. | 1066 |
| 64 | (urin* adj5 (loss* or leak* or involunt*)).tw,ot. | 6138 |
| 65 | ((urinary or emptying) adj2 (d?sfunct* or disorder* or frequency)).tw,ot. | 4443 |
| 66 | (urinary adj (symptom or symptoms)).tw,ot. | 2813 |
| 67 | urinary tract symptom*.tw,ot,kw. | 4843 |
| 68 | (urgency or nocturia or nycturia or urination or voiding or postvoid* or post-void* or urge syndrome*).tw,ot,kw. | 25865 |
| 69 | (sensation* adj2 void*).tw,ot. | 58 |
| 70 | ((nocturnal or volume) adj1 frequency).tw,ot,kw. | 552 |
| 71 | (urethra* adj3 (profilomet* or pressur*)).tw,ot. | 2202 |
| 72 | ((capacit* or overactive or over-active or hyperactive or emptying) adj3 bladder).tw,ot. | 7184 |
| 73 | detrusor overactiv*.tw,ot,kw. | 1660 |
| 74 | (SUI or UUI or OAB or LUTS).tw,ot,kw. | 9180 |
| 75 | (pad test* or bladder diar* or Q-tip or urodynamic* or uroflowmetr* or cystometr* or coughing test*).tw,ot,kw. | 13440 |
| 76 | (MUCP or MUP or PISQ or ICIQ or UDI or IIQ or PWT or CST or PTR).tw,ot,kw. | 4855 |
| 77 | (incontinen* or urinary incontinen* or stress incontin* or stress urinary incontin* or mixed incontinen* or urinary mixed incontin* or urge incontinen* or urge urin* incontin* or urgency or overactive bladder* or lower urinary tract symptom* or bothersome lower urinary tract symptom* or involuntary urin*).kw. | 955 |
| 78 | or/55-77 **[UI]** | 92493 |
| **79** | **78 and 19 [UI+ ESTR]** | **227** |
| 80 | exp Prolapse/ | 10221 |
| 81 | (prolaps* or cystoc*el* or rectoc*ele* or proctoc*ele*).tw,ot,kw. | 21164 |
| 82 | (POP-Q or POPQ or PISQ or PFDI).tw,ot,kw. | 637 |
| 83 | (bulge or bulging).tw,ot,kw. | 6226 |
| 84 | or/80-83 **[prolapse]** | 29898 |
| **85** | **84 and 19 [prolapse + ESTR]** | **90** |
