# Supplementary material for: Local Oestrogen for Pelvic Floor Disorders: A Systematic Review
Source: PLoS One. 2015 Sep 18;10(9):e0136265. doi: 10.1371/journal.pone.0136265 (PMC4575150; doi:10.1371/journal.pone.0136265)
Supplement: S2 Appendix — (DOC) [file pone.0136265.s003.doc]

**APPENDIX 2: Analysis local oestrogen for vaginal atrophy**

**Analysis 1:** Forest plot of comparison: Vaginal oestrogen tablet 0.01-0.03 mg vs. placebo, outcome: symptoms and signs after 12 months of treatment.


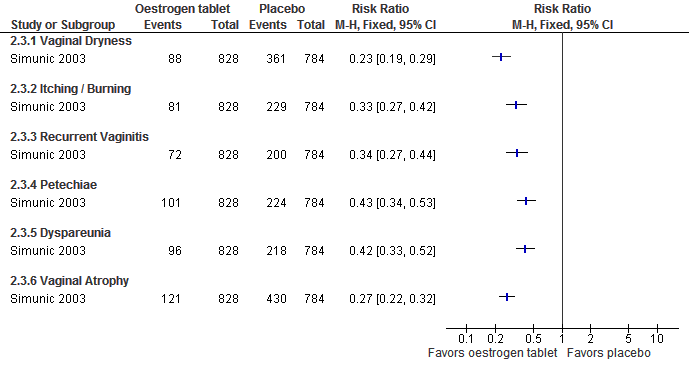


**Analysis 2:** Forest plot of comparison: Vaginal oestrogen tablet 0.01-0.03 mg vs. placebo, outcome: Total Score Index of Vaginal Atrophy after 4 months.

**
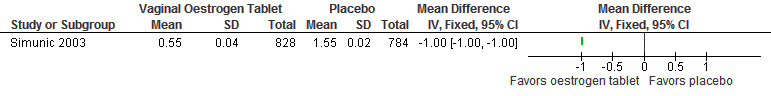
**

**Analysis 3:** Forest plot of comparison: Vaginal oestrogen tablet 0.01-0.03 mg vs. placebo, outcome: Total Score Index of Vaginal Atrophy after 12 months.

**
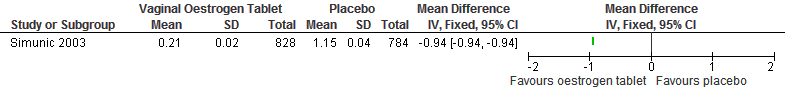
**

**Analysis 4:** Forest plot of comparison: Vaginal oestrogen ovule vs. placebo, outcome: symptoms after 6 months of treatment.

**
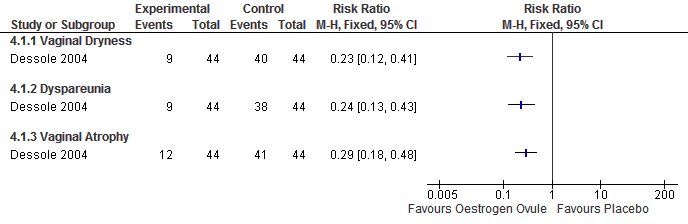
**

**Analysis 5:** Forest plot of comparison: Vaginal oestrogen tablet/pessary 0.01-0.03 mg vs. placebo, outcome: decline in MBS intensity (VAS) after 12 weeks of treatment.


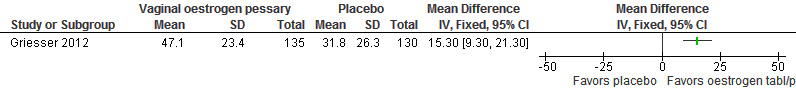


**Analysis 6:** Forest plot of comparison: Oestrogen tablet/pessary 0.2 mg vs. placebo, outcome: decline in MBS intensity (VAS) after 12 weeks of treatment.

**
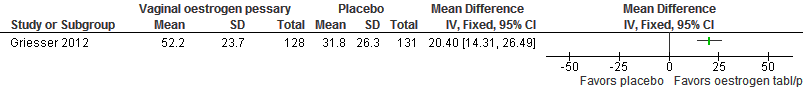
**

**Analysis 7:** Forest plot of comparison: Vaginal oestrogen cream 0,625mg vs. placebo, outcome: Urogenital score after 12 weeks of treatment.


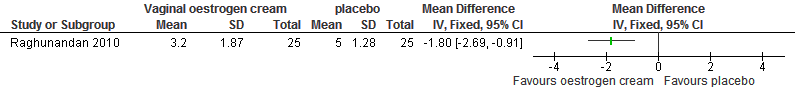


**Analysis 8:** Forest plot of comparison: Vaginal oestrogen cream 0.3-0.625 mg vs. placebo, outcome: vaginal dryness mild to moderate after 12 weeks of treatment.


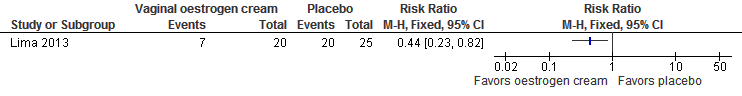


**Analysis 9:** Forest plot of comparison: Vaginal oestrogen cream 0.3-0.625 mg vs. placebo, outcome: dyspareunia mild to moderate after 12 weeks of treatment.


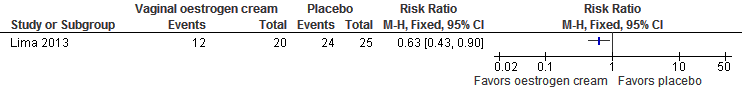


**Analysis 10:** Forest plot of comparison: Vaginal oestrogen cream 0.3-0.625 mg vs. placebo, outcome: Vaginal Health Index after 12 weeks of treatment.

**
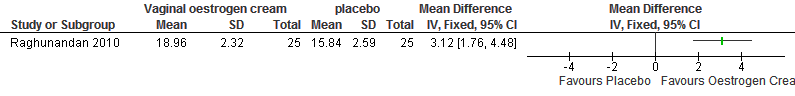
**

**Analysis 11:** Forest plot of comparison: Vaginal oestrogen ring vs. placebo, outcome: patients free of vaginal signs after 24 weeks of treatment.

**
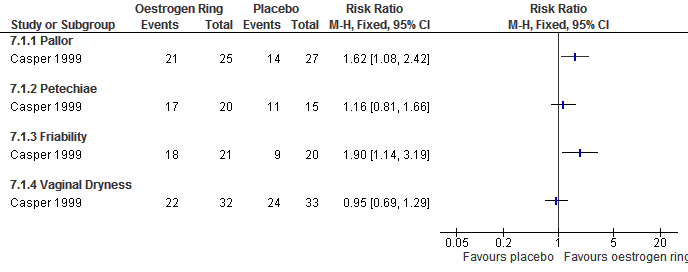
**

**Analysis 12:** Forest plot of comparison: Vaginal oestrogen cream 0,625mg vs. placebo, outcome: VMI after 12 weeks of treatment.

**
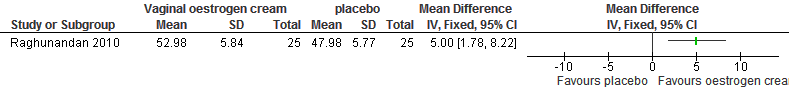
**

**Analysis 13:** Forest plot of comparison: Vaginal oestrogen ovule 1 mg vs. placebo, outcome: KPI of vaginal epithelium after 6 months of treatment.

**
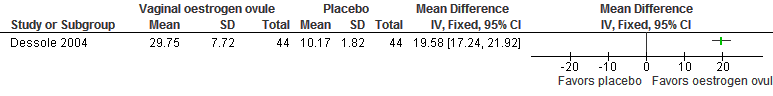
**

**Analysis 14:** Forest plot of comparison: Oestrogen gel 0,05mg vs. placebo gel, outcome: Mean change from baseline in maturation value after 12 weeks.

**
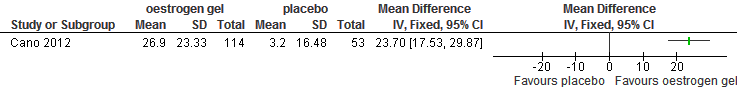
**

**Analysis 15:** Forest plot of comparison: Vaginal oestrogen tablet/pessary 0.01-0.03 mg vs. placebo, outcome: Change in VMI after 12 weeks of treatment.

**
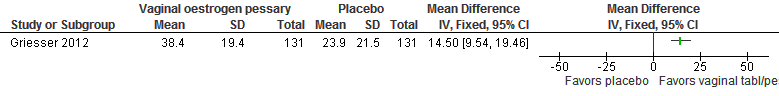
**

**Analysis 16:** Forest plot of comparison: oestrogen tablet/pessary 0.2 mg vs. placebo, outcome: Change in VMI after 12 weeks of treatment.

**
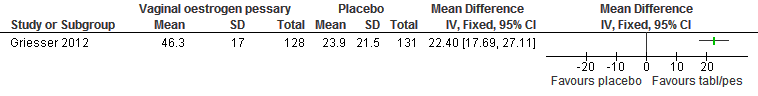
**

**Analysis 17:** Forest plot of comparison: Vaginal oestrogen ovule 1 mg vs. placebo, outcome: Vaginal pH after 6 months of treatment.

**
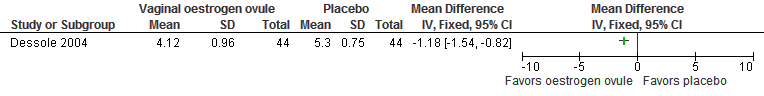
**

**Analysis 18:** Forest plot of comparison: Oestrogen gel 0,05mg vs. placebo gel, outcome: Mean change from baseline in pH after 12 weeks.

**
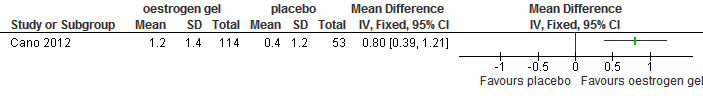
**

**Analysis 19:** Forest plot of comparison: Vaginal oestrogen tablet/pessary 0.01-0.03 mg vs. placebo, outcome: decline in pH after 12 weeks of treatment.

**
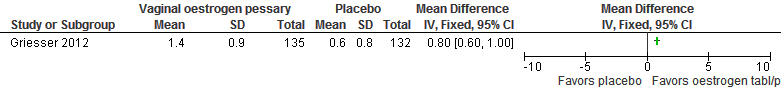
**

**Analysis 20:** Forest plot of comparison: Vaginal oestrogen tablet/pessary 0.2 mg vs. placebo, outcome: decline in vaginal pH after 12 weeks of treatment.

**
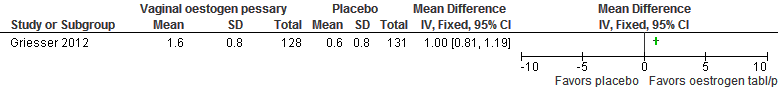
**

**Analysis 21:** Forest plot of comparison: Vaginal oestrogen tablet/pessary 0.01-0.03 mg vs. placebo, outcome: pH <5.5 after 12 weeks of treatment.

**
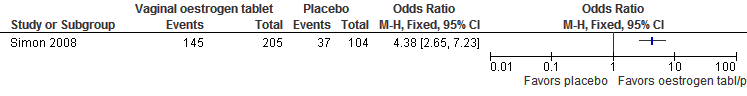
**

**Analysis 22:** Forest plot of comparison: Vaginal oestrogen tablet 0.01-0.03 mg vs. placebo, outcome: any adverse event.

**
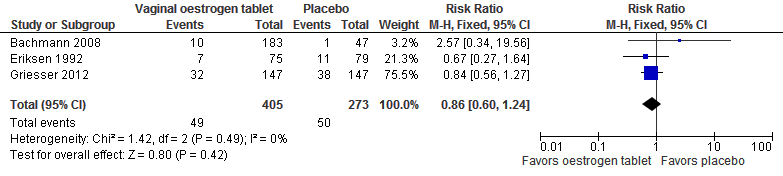
**

**Analysis 23:** Forest plot of comparison: Vaginal oestrogen cream 0.3-0.625 mg vs. placebo, outcome: adverse events.

**
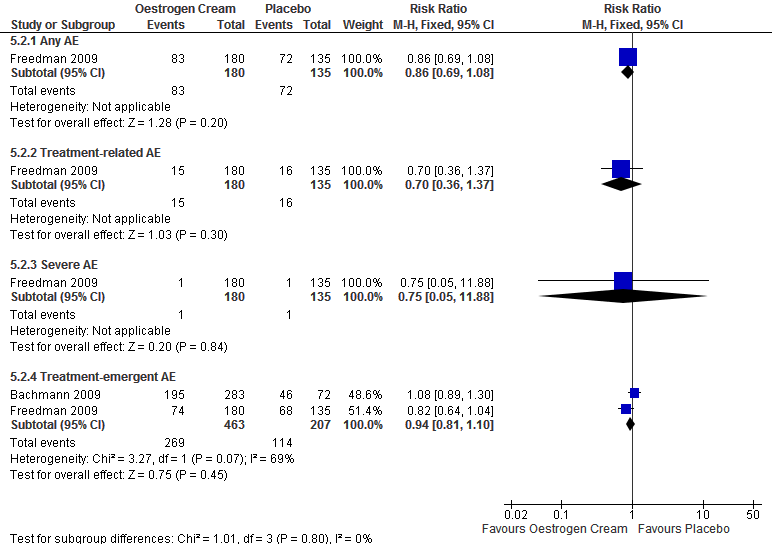
**

**Analysis 24:** Forest plot of comparison: Oestrogen gel 0,05 mg vs. placebo gel, outcome: adverse events after 12 weeks.

**
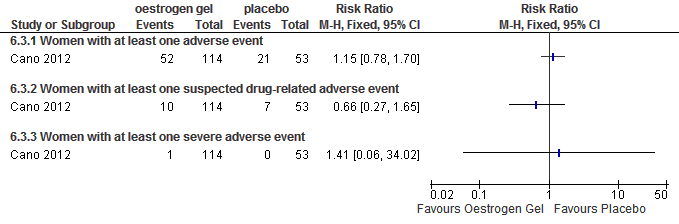
**

**Analysis 25:** Forest plot of comparison: Oestrogen pessary 0.2 mg vs. placebo, outcome: any adverse event.

**
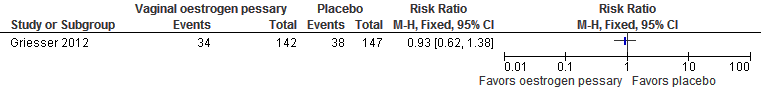
**

**Analysis 26:** Forest plot of comparison: Vaginal oestrogen tablet vs. vaginal oestrogen cream, outcome: mean score of symptoms (none (0) - severe (3)) after 12-24 weeks of treatment.

**
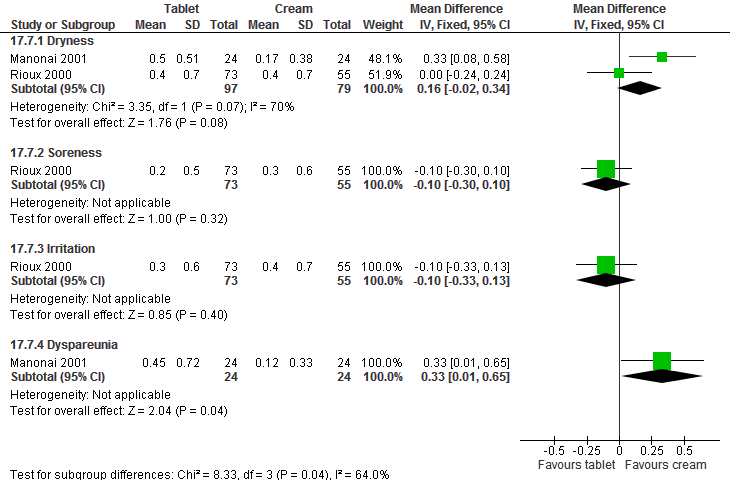
**

**Analysis 27:** Forest plot of comparison: Vaginal oestrogen ring vs. another type of application, outcome: number of patients free of symptom after 12 weeks of treatment.

**
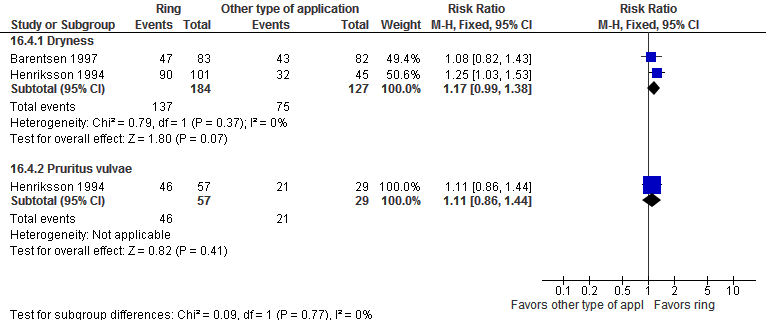
**

**Analysis 28:** Forest plot of comparison: Vaginal oestrogen ring vs. another type of application, outcome: Number of patients improved after 12 weeks of treatment.

**
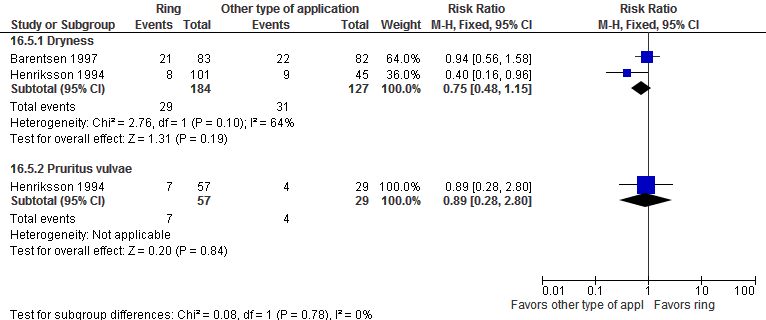
**

**Analysis 29:** Forest plot of comparison: Vaginal oestrogen ring vs. another type of application, outcome: Patient reported changes of the symptom 'vaginal dryness' after 12 weeks of treatment.

**
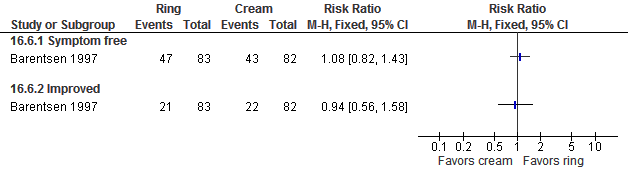
**

**Analysis 30:** Forest plot of comparison: Vaginal oestrogen ring vs. another type of application, outcome: vaginal symptoms after 48 weeks of treatment.

**
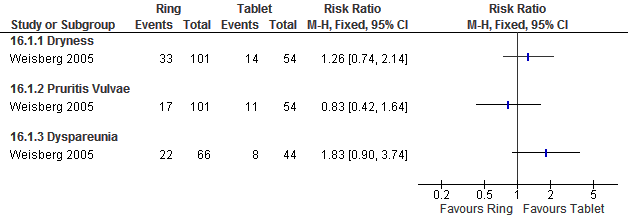
**

**Analysis 31:** Forest plot of comparison: Vaginal oestrogen tablet vs. vaginal oestrogen cream, outcome: Mean Vaginal Health Index after 12 weeks of treatment.

**
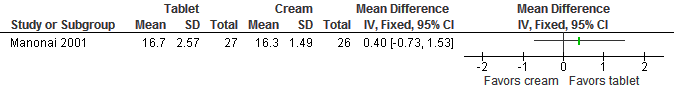
**

**Analysis 32:** Forest plot of comparison: Vaginal oestrogen ring vs. another type of application, outcome: Number of patients free of sign after 12 weeks of treatment.

**
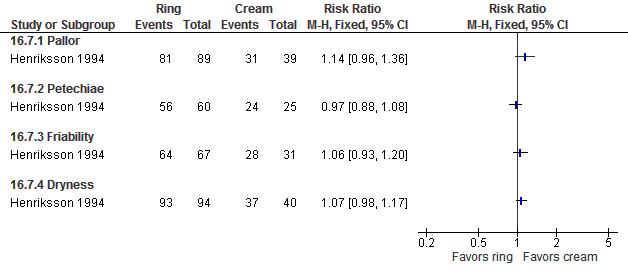
**

**Analysis 33:** Forest plot of comparison: Vaginal oestrogen tablet vs. vaginal oestrogen cream, outcome: vaginal atrophy after 24 weeks of treatment.

**
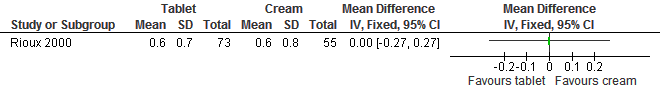
**

**Analysis 34:** Forest plot of comparison: Vaginal oestrogen ring vs. another type of application, outcome: vaginal signs after 48 weeks of treatment.

**
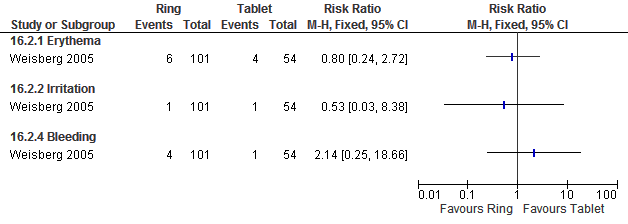
**

**Analysis 35:** Forest plot of comparison: Vaginal oestrogen ring vs. another type of application, outcome: VMV after 12 weeks of treatment.

**
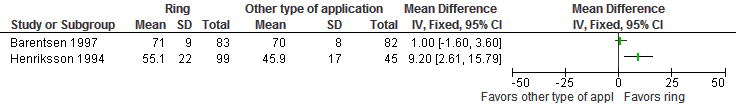
**

**Analysis 36:** Forest plot of comparison: Vaginal oestrogen ring vs. another type of application, outcome: VMI after 12 weeks of treatment.

**
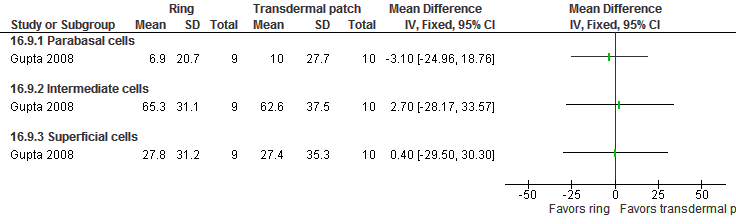
**

**Analysis 37:** Forest plot of comparison: Vaginal oestrogen ring vs. another type of application, outcome: cytological evaluation after 48 weeks of treatment.

**
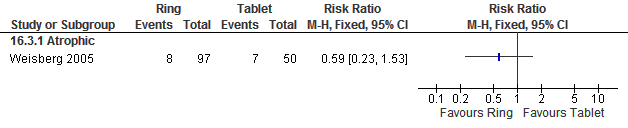
**

**Analysis 38:** Forest plot of comparison: Vaginal oestrogen tablet vs. vaginal oestrogen cream, outcome: KPI after 12 weeks of treatment.

**
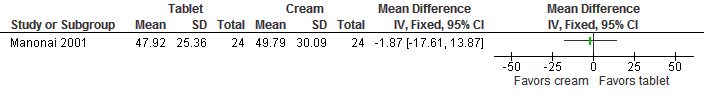
**

**Analysis 39:** Forest plot of comparison: Vaginal oestrogen ring vs. another type of application, outcome: Vaginal pH after 12 weeks of treatment.

**
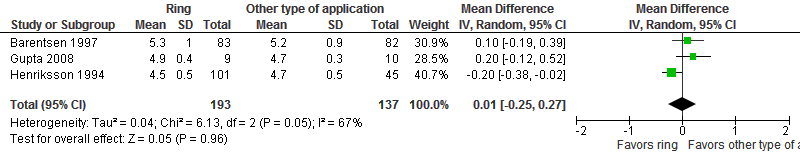
**

**Analysis 40:** Forest plot of comparison: Vaginal oestrogen tablet vs. vaginal oestrogen cream, outcome: vaginal pH after 12 weeks of treatment.

**
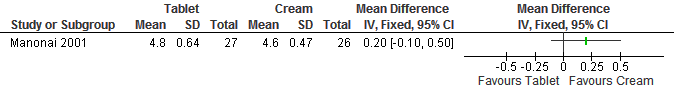
**

**Analysis 41:** Forest plot of comparison: Vaginal oestrogen ring vs. another type of application, outcome: total adverse events.

**
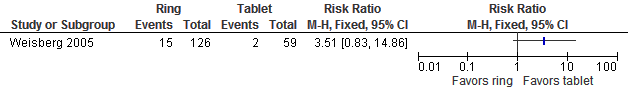
**

**Analysis 42:** Forest plot of comparison: Vaginal oestrogen cream vs. non-hormonal vaginal gel (Replens), outcome: Vaginal pH after 12 weeks of treatment*.

**
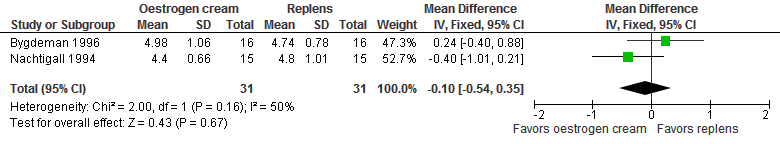
**

*Assuming Nachtigall et al. reported standard error (SE) instead of standard deviation (SD).

**Analysis 43:** Forest plot of comparison: Vaginal oestrogen cream vs. non-hormonal vaginal gel (Replens), outcome: Vaginal moisture after 12 weeks of treatment.

**
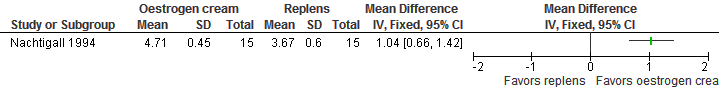
**

**Analysis 44:** Forest plot of comparison: Vaginal oestrogen cream vs. non-hormonal vaginal gel (Replens), outcome: Vaginal fluid volume after 12 weeks of treatment.

**
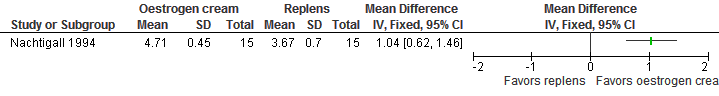
**

**Analysis 45:** Forest plot of comparison: Vaginal oestrogen cream vs. non-hormonal vaginal gel (Replens), outcome: Vaginal elasticity after 12 weeks of treatment.

**
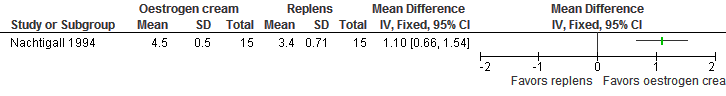
**

**Analysis 46:** Forest plot of comparison: Vaginal oestrogen cream vs. non-hormonal vaginal gel (Replens), outcome: Vaginal Dryness Index after 12 weeks of treatment.

**
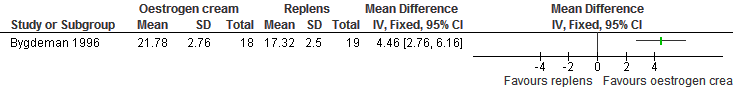
**

**Analysis 47:** Forest plot of comparison: Promestriene 1% cream (Colptrophine) vs. Estriol 0.1% cream (Ovestrion), outcome: atrophic.


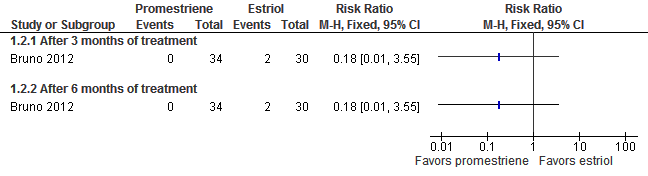


**Analysis 48:** Forest plot of comparison: Vaginal oestrogen tablet 0.025 mg vs. vaginal oestrogen vagitory 0.5 mg, outcome: cytology results.

**
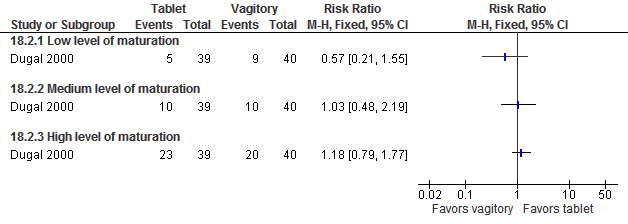
**

**Analysis 49:** Forest plot of comparison: Vaginal oestrogen tablet 0.025 mg vs. vaginal oestrogen vagitory 0.5 mg, outcome: total adverse events.

**
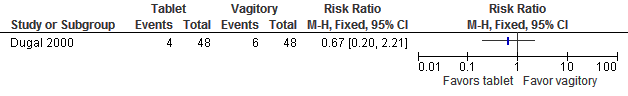
**

**Analysis 50:** Forest plot of comparison: Vaginal oestrogen ovule vs. vaginal oestrogen ovule and pelvic floor rehabilitation, outcome: vaginal dryness after 6 months of treatment.

**
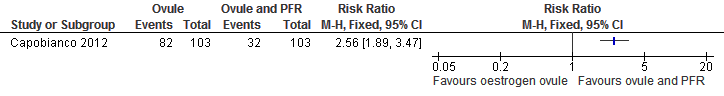
**

**Analysis 51:** Forest plot of comparison: Vaginal oestrogen ovule vs. vaginal oestrogen ovule and pelvic floor rehabilitation, outcome: dyspareunia after 6 months of treatment.

**
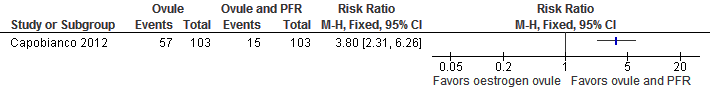
**

**Analysis 52:** Forest plot of comparison: Triple therapy (oestrogen and Lactobacilli Acidophili ovule plus pelvic floor rehabilitation (PFR)) vs. oestrogen ovule plus PFR, outcome: vaginal dryness after 6 months of treatment.

**
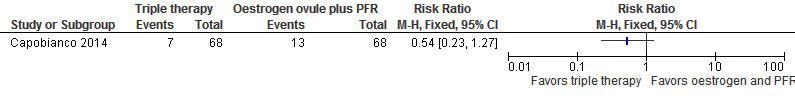
**

**Analysis 53:** Forest plot of comparison: Triple therapy (oestrogen and Lactobacilli Acidophili ovule plus pelvic floor rehabilitation (PFR)) vs. oestrogen ovule plus PFR, outcome: dyspareunia after 6 months of treatment.

**
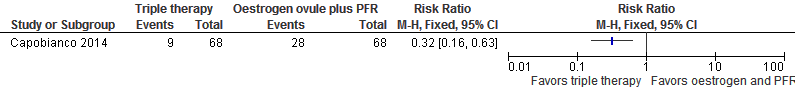
**

**Analysis 54:** Forest plot of comparison: Vaginal oestrogen ovule vs. vaginal oestrogen ovule and Pelvic Floor Rehabilitation, outcome: vaginal atrophy after 6 months of treatment.

**
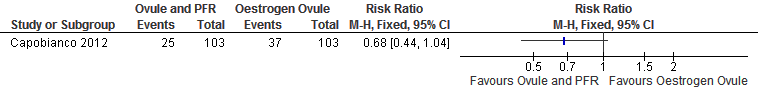
**

**Analysis 55:** Forest plot of comparison: Triple therapy (oestrogen and Lactobacilli Acidophili ovule plus PFR) vs. oestrogen ovule plus PFR, outcome: vaginal atrophy after 6 months of treatment.

**
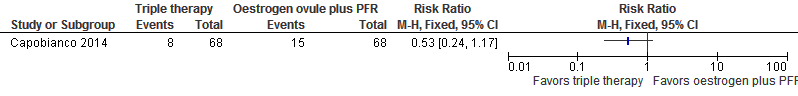
**

**Analysis 56:** Forest plot of comparison: Vaginal oestrogen ovule vs. vaginal oestrogen ovule and Pelvic Floor Rehabilitation, outcome: KPI (karyopyknotic index) of vaginal epithelium after 6 months of treatment.

**
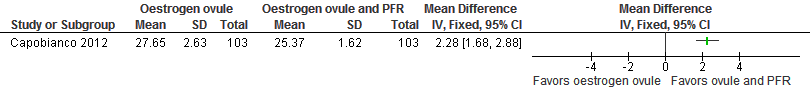
**

**Analysis 57:** Forest plot of comparison: Triple therapy (oestrogen and Lactobacilli Acidophili ovule PFR) vs. oestrogen ovule plus PFR, outcome: KPI (karyopyknotic index) of vaginal epithelium after 6 months of treatment.

**
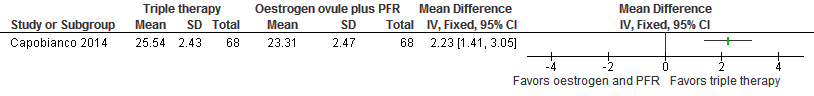
**

**Analysis 58:** Forest plot of comparison: Vaginal oestrogen ovule vs. vaginal oestrogen ovule and Pelvic Floor Rehabilitation, outcome: Vaginal pH after 6 months of treatment.

**
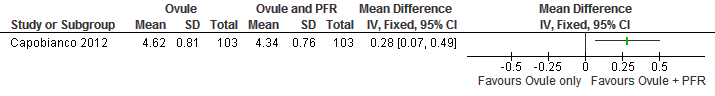
**

**Analysis 59:** Forest plot of comparison: Triple therapy (oestrogen and Lactobacilli Acidophili ovule plus PFR) vs. oestrogen ovule plus PFR, outcome: pH after 6 months of treatment.

**
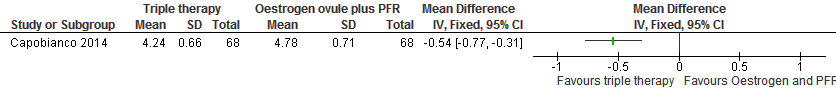
**

**Analysis 60:** Forest plot of comparison: Vaginal oestrogen cream vs. vaginal oestrogen cream and testosterone cream, outcome: Urogenital Score after 12 weeks of treatment.

**
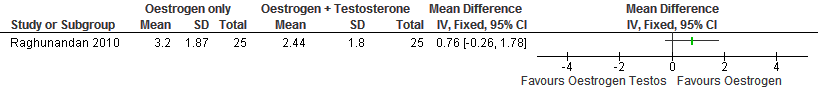
**

**Analysis 61:** Forest plot of comparison: Vaginal Oestrogen Cream vs. vaginal oestrogen cream and testosterone cream, outcome: Vaginal Health Index after 12 weeks of treatment.

**
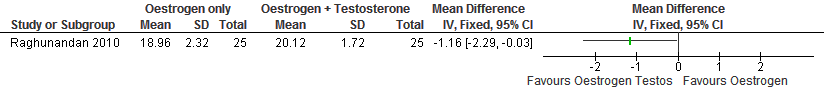
**

**Analysis 62:** Forest plot of comparison: Vaginal oestrogen cream vs. vaginal oestrogen cream and testosterone Cream, outcome: VMI after 12 weeks of treatment.

**
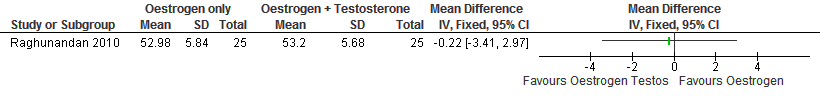
**
