# Supplementary material for: Local Oestrogen for Pelvic Floor Disorders: A Systematic Review
Source: PLoS One. 2015 Sep 18;10(9):e0136265. doi: 10.1371/journal.pone.0136265 (PMC4575150; doi:10.1371/journal.pone.0136265)
Supplement: S3 Appendix — (DOC) [file pone.0136265.s004.doc]

**APPENDIX 3: Analysis local oestrogen for urinary incontinence and OAB**

**Analysis 1:** Forest plot of comparison: Vaginal oestrogen vs. placebo, outcome: symptoms after treatment.

**
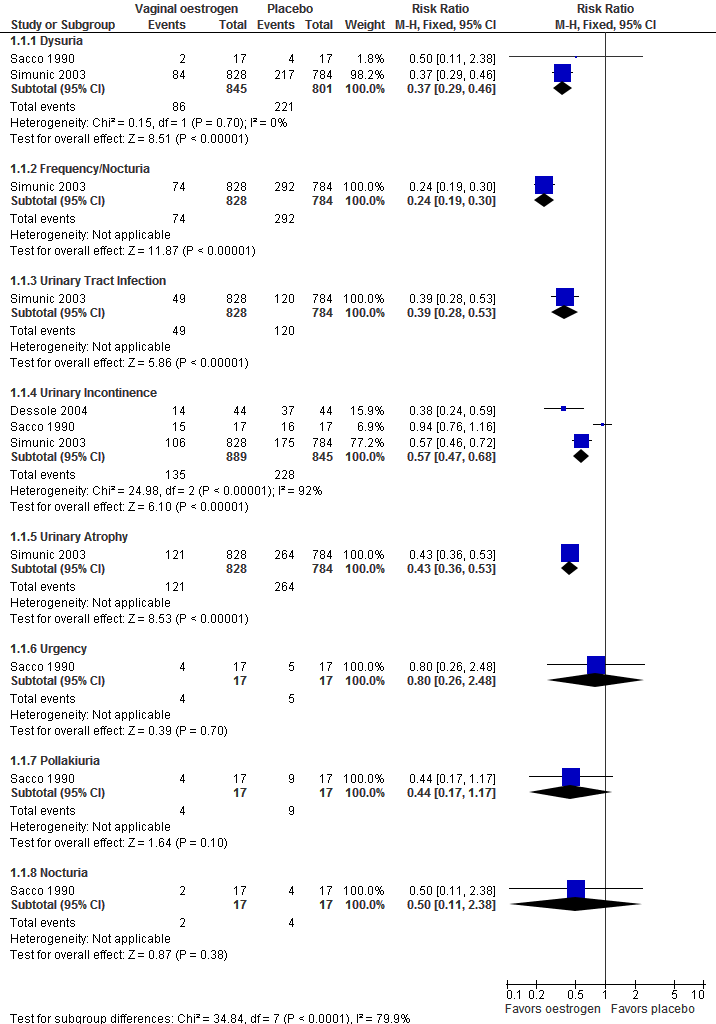
**

**Analysis 2:** Forest plot of comparison: Vaginal oestrogen vs. placebo, outcome: symptoms after treatment.

**
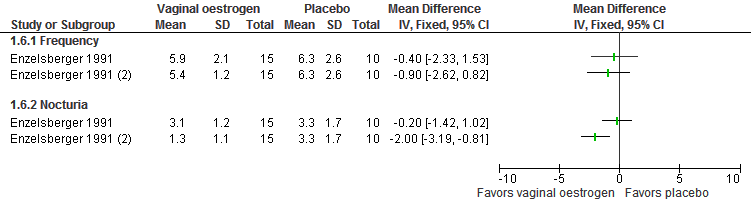
**

**Analysis 3:** Forest plot of comparison: Premarin cream vs. no treatment, outcome: unchanged based on pad weight changes.

**
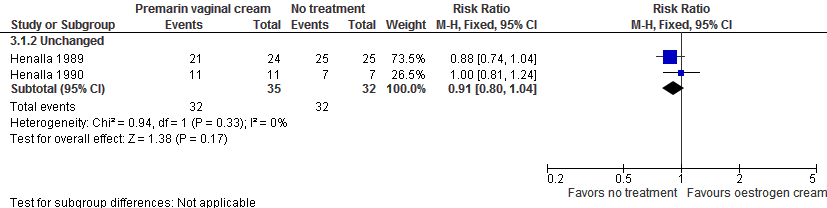
**

**Analysis 4:** Forest plot of comparison: Vaginal oestrogen vs. placebo, outcome: urodynamic parameters after treatment.

**
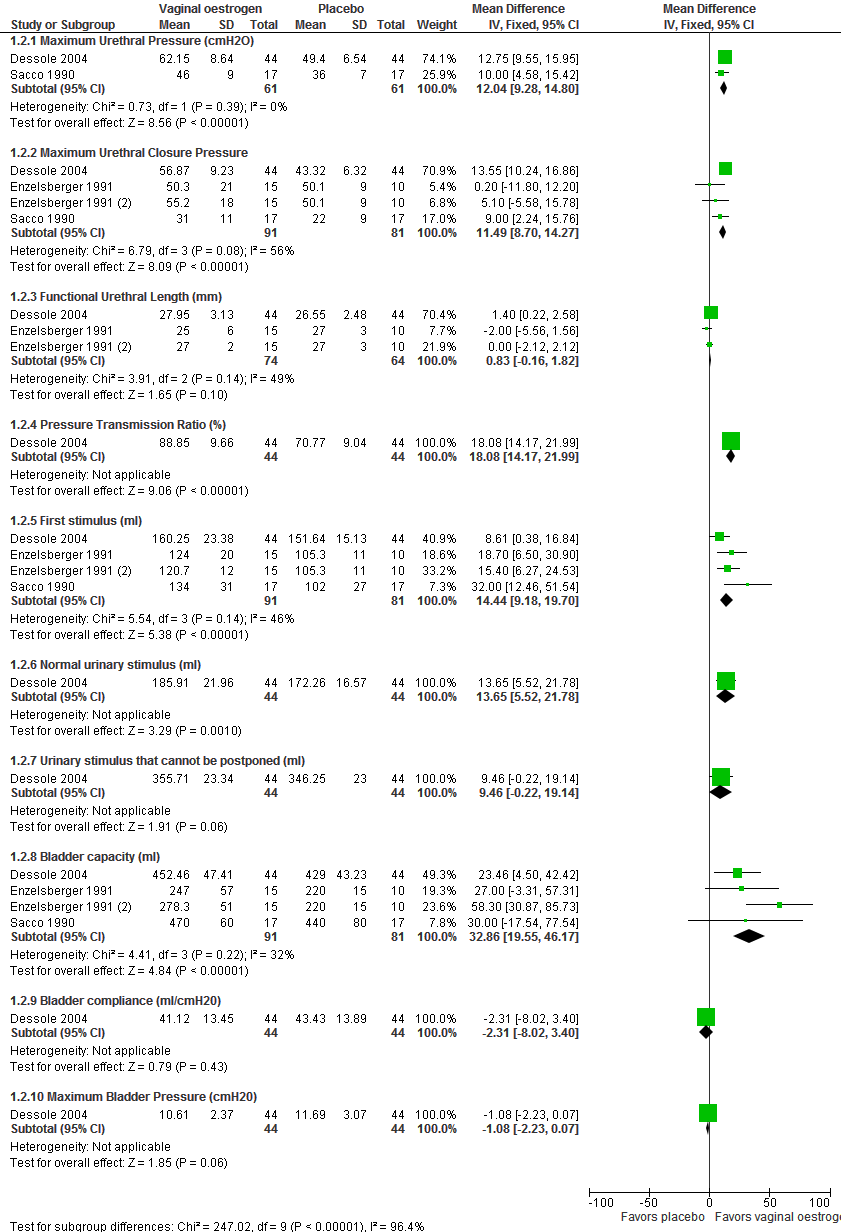
**

**Analysis 5:** Forest plot of comparison: Oestrogen ring vs. oestrogen pessary, outcome: subjective judgment / overall assessment after 24 weeks of treatment.

**
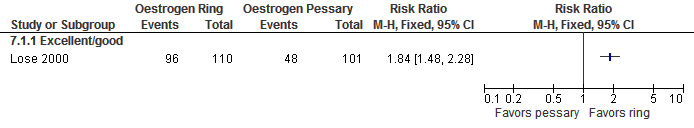
**

**Analysis 6:** Forest plot of comparison: Oestrogen ring vs. oestrogen pessary, outcome: number with adverse events.

**
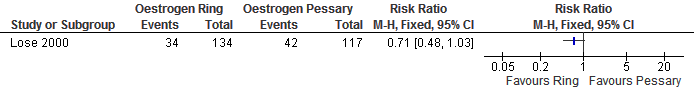
**

**Analysis 7:** Forest plot of comparison: Tolterodine plus vaginal oestrogen cream vs. tolterodine only, outcome: efficacy after 12 weeks of treatment.

**
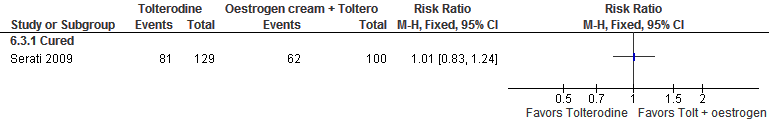
**

**Analysis 8:** Forest plot of comparison: Tolterodine plus vaginal oestrogen cream vs. tolterodine only, outcome: UDI-6 score after 12 weeks of treatment*.

**
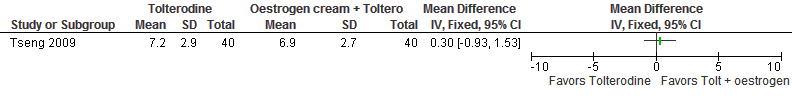
**

**Analysis 9:** Forest plot of comparison: Tolterodine plus vaginal oestrogen cream vs. tolterodine only, outcome: IIQ-7 score after 12 weeks of treatment*.

**
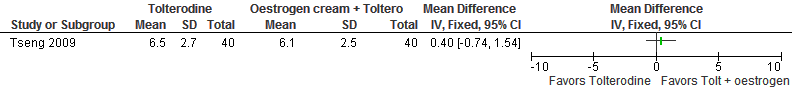
**

*****Tseng and co-workers reported that the tolterodine/oestrogen group had a statistically significant decrease in the UDI-6 and IIQ-7 scores than that decreased in the tolterodine alone group. However, when extracting these data in analysis 8 and 9 differences in end scores do not seem statistically significant. Women in the tolterodine group had higher UDI-6 scores at baseline.

**Analysis 10:** Forest plot of comparison: Oestrogen vaginal ring vs. oral oxybutynin, outcome: Urogenital Distress Inventory (UDI-6 score) after 12 weeks of treatment.


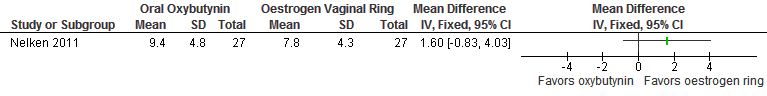


**Analysis 11:** Forest plot of comparison: Oestrogen vaginal ring vs. oral oxybutynin, outcome: Incontinence Impact Questionnaire (IIQ-7 score) after 12 weeks of treatment.

**
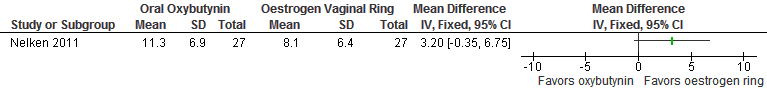
**

**Analysis 12:** Forest plot of comparison: Tolterodine plus vaginal oestrogen cream vs. tolterodine only, outcome: bladder diary variables after 12 weeks of treatment.

**
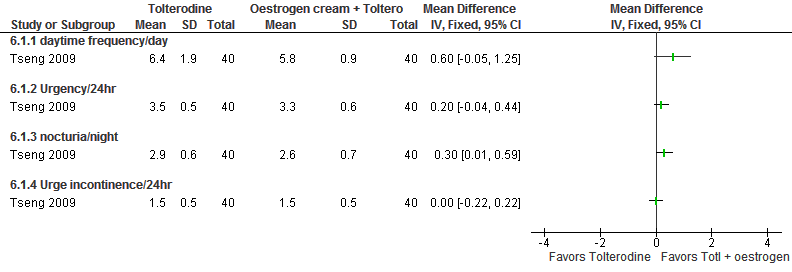
**

**Analysis 13:** Forest plot of comparison: Tolterodine plus vaginal oestrogen cream vs. tolterodine only, outcome: Voided volume after 12 weeks of treatment.

**
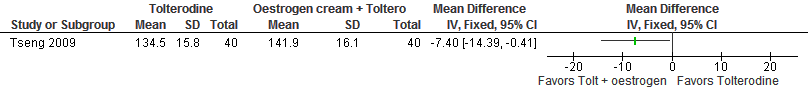
**

**Analysis 14:** Forest plot of comparison: Oestrogen vaginal ring vs. oral oxybutynin, outcome: mean number of voids after 12 weeks of treatment.

**
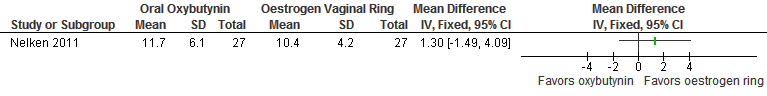
**

**Analysis 15:** Forest plot of comparison: Premarin vaginal cream vs. pelvic floor exercises, outcome: Cured and improved based on pad weight changes.

**
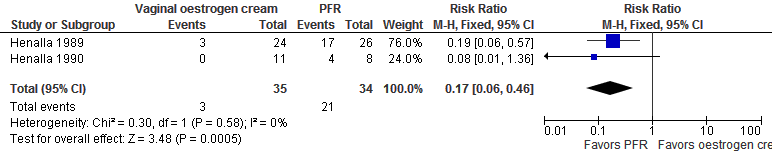
**

**Analysis 16:** Forest plot of comparison: Premarin vaginal cream vs. pelvic floor electrostimulation, outcome: Cured and improved based on pad weight changes.


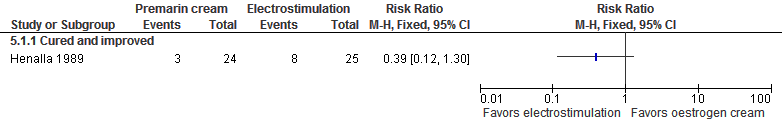


**Analysis 17:** Forest plot of comparison: Oestrogen vaginal ring vs. oral oxybutynin, outcome: adverse events after 12 weeks of treatment.

**
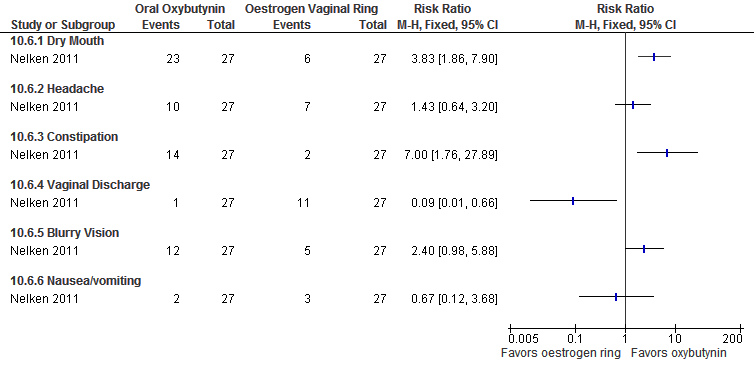
**

**Analysis 18:** Forest plot of comparison: 0,5/1mg oestrogen vs. 2 mg oestrogen, outcome: bladder diary variables after 3 weeks of treatment.

**
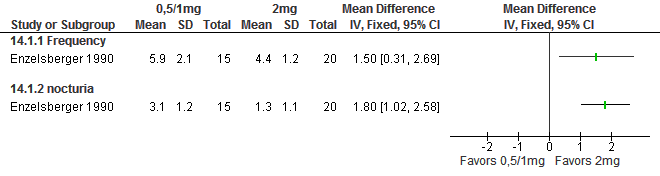
**

**Analysis 19:** Forest plot of comparison: 1 mg oestrogen vs. 3 mg oestrogen, outcome: bladder diary variables after 3 weeks of treatment.

**
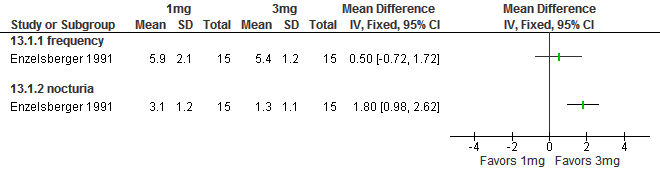
**

**Analysis 20:** Forest plot of comparison: 0,5/1mg oestrogen vs. 2 mg oestrogen, outcome: urodynamic variables after 3 weeks of treatment.

**
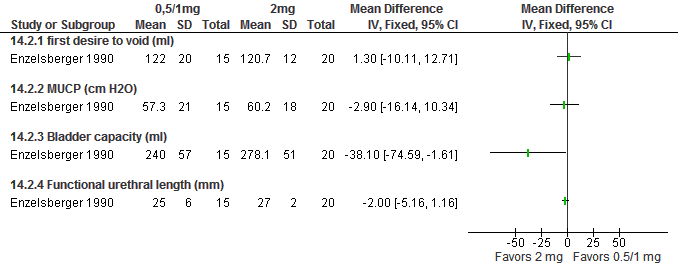
**

**Analysis 21:** Forest plot of comparison: 1 mg oestrogen vs. 3 mg oestrogen, outcome: urodynamic variables after 3 weeks of treatment.

**
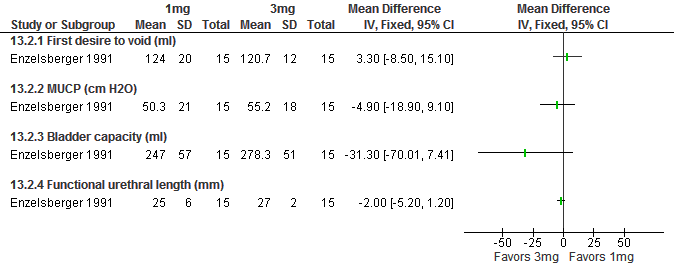
**

**Analysis 22:** Forest plot of comparison: Vaginal oestrogen cream vs. oral oestrogen, outcome: urinary symptoms after 3 months of treatment.

**
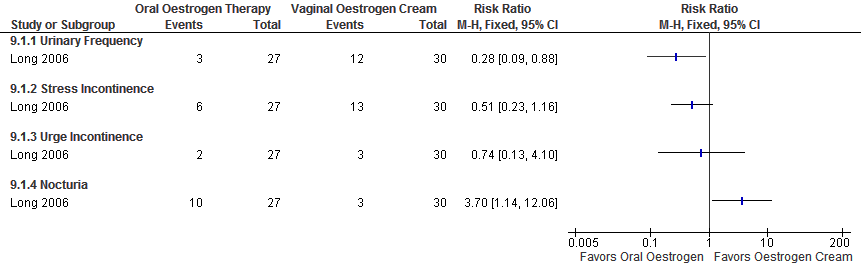
**

**Analysis 23:** Forest plot of comparison: Vaginal oestrogen cream vs. oral oestrogen, outcome: SUI episodes per week in improved patients.

**
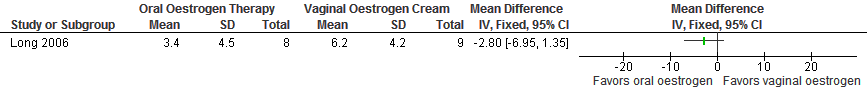
**

**Analysis 24:** Forest plot of comparison: Oestrogen Ovule plus PFR vs. oestrogen ovule only, outcome: clinical outcomes after 6 months of treatment.

**
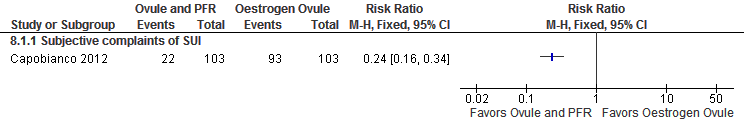
**

**Analysis 25:** Forest plot of comparison: Triple therapy (vaginal oestrogen ovule and Lactobacilli acidophili plus PFR) vs. vaginal oestrogen ovule and PFR, outcome: clinical variables after 6 months of treatment.

**
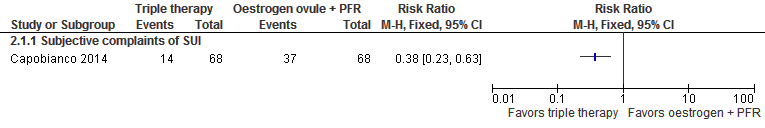
**

**Analysis 26:** Forest plot of comparison: Oestrogen Ovule plus PFR vs. oestrogen ovule only, outcome: urodynamic variables after 6 months of treatment.

**
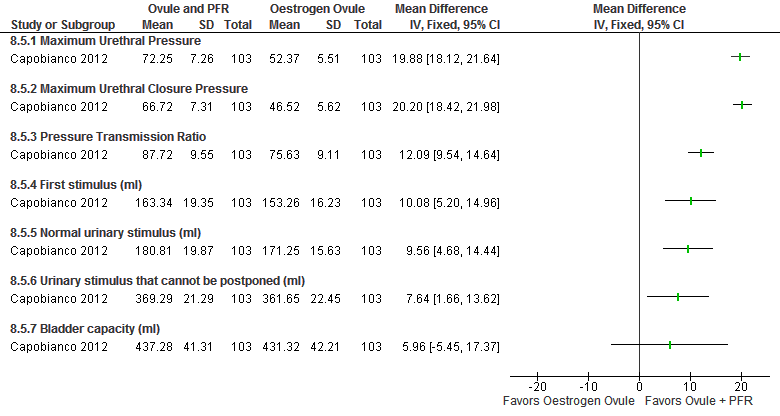
**

**Analysis 27:** Forest plot of comparison: Triple therapy (vaginal oestrogen ovule and Lactobacilli acidophili plus PFR) vs. vaginal oestrogen ovule plus PFR, outcome: urodynamic variables after six months of treatment

**
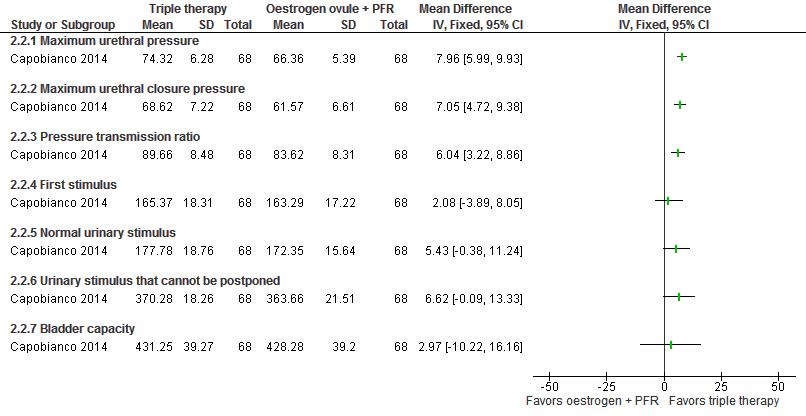
**
